# Recommendations for Research and Clinical Implementation of Ambulatory Assessment, Mood Monitoring, Digital Phenotyping, and Remote Measurement Technology in Mood Disorders: Synthesis of Systematic Review Findings

**DOI:** 10.2196/79501

**Published:** 2026-06-02

**Authors:** Laurence Astill Wright, Mat Rawsthorne, Neil Nixon, Boliang Guo, Richard Morriss

**Affiliations:** 1 Institute of Mental Health, University of Nottingham, University of Nottingham Innovation Park Jubilee Campus, Triumph Road, Nottingham, NG7 2TU, United Kingdom, 44 0115 823 1294; 2 Centre for Academic Mental Health, Population Health Sciences, University of Bristol, Bristol, United Kingdom; 3 NIHR ARC East Midlands, University of Nottingham, Nottingham, United Kingdom; 4 Nottingham NIHR Biomedical Research Centre, University of Nottingham, Nottingham, United Kingdom; 5 NIHR MindTech Medical Technology Collaborative, University of Nottingham, Nottingham, United Kingdom

**Keywords:** depression, bipolar, ambulatory assessment, ecological momentary assessment, EMA, mood-tracking, mood-monitoring, self-monitoring, adverse effects, real-world evidence, digital phenotyping, remote measurement technology, passive monitoring

## Abstract

**Background:**

Ambulatory assessment and active and passive monitoring all offer a real-time, flexible approach to assessing mood and behavior in mood disorders. Despite their potential, concerns remain regarding the performance, usability, adherence, and potential safety of these tools.

**Objective:**

This study synthesizes the findings from 7 systematic reviews, integrating quantitative and qualitative data from randomized trials, observational studies, and user experience research to evaluate the performance, feasibility, acceptability, and clinical impact of ambulatory assessment and mood monitoring in people with depression and bipolar disorder. We assessed studies over the medium or long term (3 months or more).

**Methods:**

A summary of a series of systematic reviews was carried out by the authors—including meta-analyses (for quantitative data) and meta-syntheses (for qualitative data). Eight electronic databases were searched, and mixed methods studies were included. Studies were assessed for risk of bias. The results were checked for coherence, and recommendations were made by individuals with lived experience, methodologists, and psychiatrists. GRADE (Grading of Recommendations Assessment, Development, and Evaluation) was used to assess the quality and strength of the evidence.

**Results:**

The 111 included studies included 19,945 participants and used 69 different ambulatory assessment protocols or mood-monitoring interventions. Key barriers to implementation were identified, including performance inconsistency, adverse effects, and user disengagement. Evidence-based recommendations are provided to guide future clinical and research applications.

**Conclusions:**

Ambulatory assessment and mood monitoring hold promise in research and clinical practice, yet their implementation requires more rigorous evaluation, greater personalization, and responsible, user-centered design. Crucially, these measures can add granularity and confirmation, but additional context is often required, and none of these measures are robust enough yet to replace current outcomes.

## Introduction

This synthesis of systematic reviews evaluates medium- or long-term (3 months or more) ambulatory assessment and mood monitoring protocols in depression and bipolar disorder (BD). It provides evidence-based recommendations for clinical and research use based on effectiveness, performance, and user experience. These recommendations serve as a key blueprint for the design and implementation of future ambulatory assessment protocols.

First, we define the different types of ambulatory assessment discussed in this review and the different use cases of these types. Ambulatory assessment includes ecological momentary assessment (EMA—more intensive self-report eg, multiple times per day [1,2]), remote measurement technology (eg, wearables to passively collect data in the background), and mood monitoring or mood tracking. This review assesses mood monitoring studies, and some of these studies also fall under EMA, remote measurement technology, and ambulatory assessment studies, but there is often overlap in their definitions. Mood monitoring can be used as a research measure (both in randomized controlled trials [RCTs] and nonrandomized studies) and as an intervention (both in RCTs and nonrandomized studies)—both in potentially highly personalized ways that offer improvements in efficiency, flexibility, and usability [3,4]. Mood monitoring can be active or passive. Passive monitoring does not require input from the user to collect data and operates in the background. Passive monitoring is less intrusive and occurs automatically but often lacks granularity [5] because the interpretation of these measures requires additional context (eg, if the individual is experiencing agitation or just taking a walk in the case of step counters). These measures of objective behavior or physiology, however, do not explore the subjective experience of mood [6]. Active monitoring (requiring the participant to actively input data) takes effort on the part of the participant, adds granularity to passive measures, and can be completed when convenient for the participant [7]. EMA is perceived as more intrusive because it requires responses in the moment, sometimes when it is not convenient. EMA does not provide a snapshot over time unless the data are aggregated—it captures subjective experience but does not fully explore the content because it is brief and occurs in the moment [1,8]. These distinct tools all have the potential to complement each other when used in the right way. Furthermore, using modern techniques, it is common for EMA to assess multiple aspects of experience at the same time, for example, location, social context, activities, mood, and psychological symptoms in a single survey [9]. This density of sampling determines content coverage, and if participants are sampled several times a day, a wide range of information can be collected, not just information related to mood and psychological symptoms [8]. In this study, we evaluate and discuss these different use cases.

This review is particularly important as the interest in ambulatory assessment approaches in mental health has increased substantially in recent years. This is due to the development of new technologies that may be key in delivering new mental health treatments and collecting large volumes of research data that can potentially capture the dynamic fluctuations in psychopathology and behavior that traditional measures or interventions can struggle to capture [10]. These fluctuations are associated with mental relapse, recurrence, wellness, reliable recovery, distress, and risk-taking behaviors [11]. Ambulatory assessment offers the possibility of detecting mood instability earlier and more continuously than traditional methods. The measurement of these fluctuations may improve personalized prediction and prognosis as well as treatment outcomes via digital phenotyping [12]. Ambulatory assessment approaches may also be more convenient. For example, while they may avoid the felt stigma of certain engagements with mental health services (eg, attending a clinic) and avoid language-based complications (eg, translation), they do, however, often require significant motivation on the part of the participant.

While ambulatory assessment and mood monitoring approaches hold promise, current evidence reveals mixed performance, uncertain efficacy, and complex user experiences [13]. On the one hand, mood tracking via new technologies (such as wearable devices and smartphone sensors) is broadly liked by people with mood disorders and is often used to support well-being [14,15]. On the other hand, there are concerns within the field regarding the implementation of these technologies [16,17]. Some of these concerns include the performance of the measures (eg, do they measure what they claim to?), their usability and acceptability, the possibility of adverse events and negative psychological impacts, and whether they exert any direct effect on mood or not (eg, will they make effective interventions for people with mental disorders?) [13-15]. We sought to provide key answers to these concerns by reviewing the data—specifically focusing on studies that were closer to clinical implementation (eg, with follow-up periods of a minimum of 3 months).

Here, we summarize the results of a series of systematic reviews and provide evidence-based recommendations for the development of future ambulatory assessment protocols or mood monitoring interventions. We treat these 2 functions—one as the gathering of data for research assessment and the other as the gathering of data to deliver a personalized intervention (eg, just-in-time adaptive interventions [18])—as 2 distinct purposes; therefore, the recommendations or considerations are separate and different in nuanced ways. In this review, we included studies that have assessed mood at least weekly, with most ambulatory assessment protocols using daily, multiple-times-per-day, or continuous assessment. The frequency of assessment depended on the purpose of the study and the behavior assessed (eg, continuous step count monitoring vs visual analog scale of mood).

The majority of the reviews reported here have been published, and there was not sufficient space in these studies to fully detail these important recommendations to the field—both for future interventions and research measures. We present this as a separate paper to fully discuss the implications of our research for the future development of these technologies. This is a common approach that expands on already published data by providing a broader expert consensus, which provides important context to the findings of the 7 individual papers. This does not, however, include any new unpublished data (or data currently in peer review—one paper).

## Methods

### Recommendations and Grading of the Evidence

Evidence-based recommendations and grading of the strength of the evidence for each recommendation were developed up by a multidisciplinary group involving psychiatrists, methodologists, and service users who were involved in the original design, data analysis, and subsequent interpretation of the reviews. We used GRADE (Grading of Recommendations Assessment, Development and Evaluation) to assess the quality of the evidence and the strength of the recommendations (high, moderate, low, or very low) [19].

### Methodology

This paper itself is not a review but synthesises the findings of a series of reviews, the methodology of which is discussed here.

We include the eligibility criteria here as these are important for appraising the recommendations we make in this paper. We included studies that met the following criteria: (1) quantitative studies including self-monitoring, EMA, or repeated symptom assessment in people with depression or BD using an interventional design over a minimum period of 3 months, with symptoms rated weekly at a minimum. We set this minimum period of 3 months of use to include studies that were closer to real-world implementation and because we reasoned that 3 months of use was the minimum requirement for many people with BD or depression to observe a significant change in mood. Three months is also a frequent primary outcome assessment point for many key interventional studies. (2) Qualitative studies exploring user perspectives on self-monitoring or ambulatory assessment or repeated symptom assessment in people with depression or BD. We defined depression as a current or previous clinical diagnosis, a self-reported diagnosis, or meeting research criteria for a depressive disorder. Self-reported depressive symptoms and depression in the context of BD or depression were excluded, as the user experience was theorized to be distinct. We included actual use and hypothetical use of ambulatory assessment or self-monitoring for depressive symptoms. The studies could be published in any language and could involve digital or nondigital methods.

## Results

RCTs and nonrandomized studies used ambulatory assessment or mood tracking as an outcome or an intervention. Qualitative studies explored the user experience of ambulatory assessment or mood tracking protocols, or used coproduction or implementation frameworks to improve the usability and acceptability of the protocol. Table 1 outlines the overall findings from all the systematic reviews.

The search identified 23,515 papers. A total of 111 papers met the eligibility criteria and were included in the series of systematic reviews of which this paper is a summary.

The 111 included studies were performed on 19,945 participants and used 69 different ambulatory assessment protocols or mood monitoring interventions. Table S1 in Multimedia Appendix 1 displays detailed characteristics of the studies and the ambulatory assessment protocols used. We included 34 qualitative studies, 28 RCTs, and 49 nonrandomized studies. Follow-up periods varied from 3 months to 3 years. All studies included participants with depression or BD; however, 10 studies used mixed samples [20-28]. Four studies used samples of young people or adolescents [29-32], while the rest used adult samples.

RCTs and nonrandomized studies used ambulatory assessment or mood tracking as either an outcome or an intervention. Qualitative studies explored the user experience of ambulatory assessment or mood tracking protocols or used coproduction or implementation frameworks to improve the usability and acceptability of the protocol. Table 1 outlines the overall findings from all systematic reviews.

The evidence-based recommendations from our research are summarized in Table 2, along with the strength of evidence supporting them, as assessed using GRADE [19].

**Table 1.** Summary of key findings across systematic reviews of qualitative and quantitative studies.

Review	Key findings				
	Performance	Adverse effects	User preferences	Clinical effectiveness	Design considerations
User experience—depression	Limited discussion; performance concerns were raised by participants.	Mood monitoring can be confronting and stressful; some participants felt burdened; participants experienced technical problems.	Participants expressed a strong desire for simplicity; passive data approaches were preferred; a focus on positives rather than negatives was preferred; participants wanted their data integrated with their health care.	Participants voiced mixed views on whether it was effective for them; some felt that effectiveness was reliant on integration with their mental health care.	Participants prioritized ease of use; wished for positive feedback and for a passive data-led protocol.
User experience—BD^a^	Not reported.	People with BD raised concerns over intrusive features of the protocols, for example, notifications. Some reported a worsening of their mood through use.	There was a strong emphasis on retaining control over data, personal autonomy, and using the tool outside of conventional mental health services.	People with BD used the tools for their self-management of their illness, not for symptom reduction.	Participants thought that customization of the tool was critical. They felt that clinician involvement was optional and often undesired.
RCTs^b^ depression and BD	Active ambulatory assessment methods show good performance (concurrent validity) in trials; passive ambulatory assessment demonstrates inconsistent performance.	This was underreported across RCTs.	Not reported.	There was no clear evidence of symptom improvement.	There is a need for high-quality trials that evaluate only mood monitoring without additional therapeutic elements.
Depression performance	Passive ambulatory assessment performed poorly (weak correlation coefficients); active ambulatory assessment may have stronger performance.	Not reported.	Not reported.	Not reported.	Future studies should consider the standardization of performance metrics to enable comparison with established measures.
BD performance	Active ambulatory assessment measures demonstrated good performance. There were sparse data assessing passive ambulatory assessment measures.	Not reported.	Not reported.	Not reported.	Future studies should ensure greater consistency in ambulatory assessment design and reporting standards.
Adverse events	Not reported.	Approximately 2% reported mood worsening; 4% burden/stress; 5% self-harm.	Mood monitoring was helpful and easy to use; however, it was time-consuming, and there were frequent technical issues. Personalization was suggested as an improvement.	The risk of harm needs to be considered in implementation and protocol design.	More systematic monitoring of adverse events is needed to optimize safety and usability.
Attrition and adherence	Not reported.	Adherence was low at 64%, and attrition was high at 28%. This may suggest usability issues or adverse events.	Not reported.	Improving attrition and adherence is likely to improve clinical effectiveness.	Reporting of attrition and adherence should be universal and systematic.

a BD: bipolar disorder.

b RCT: randomized controlled trial.

**Table 2.** Evidence-based recommendations for ambulatory assessment and mood monitoring in mood disorders.

Domain	Recommendation	Grade of supporting evidence
Mood monitoring interventions		
Target population	Prioritize asymptomatic individuals at risk of relapse, especially those with high insight.	Very low
Purpose	Focus on self-management, relapse prevention, staying well, and insight rather than symptom reduction alone.	High
Personalization	Enable users to customize data collection (type, frequency), data sharing, notifications, and feedback.	High
Therapeutic integration	Support optional integration with clinicians—recognizing distinct preferences in BD^a^ versus depression.	Moderate
Adverse effects	Screen and monitor for potential harms (eg, mood worsening, stress, rumination).	Moderate
Feedback design	Co-design feedback with users to optimize acceptability, for example, tone of feedback.	High
Technological reliability	Minimize technological problems and usability issues to maximize engagement.	High
Onboarding	Provide initial support to help users interpret data and set goals.	Very low
Ambulatory assessment protocols for research		
Outcome consistency	Use standardized measures of ambulatory assessment performance and report in main study paper.	High
Data collection	Passive ambulatory assessment is preferred, if the performance is good. This may need to be supplemented by active ambulatory assessment to validate the findings.	Moderate
User burden	Reduce participant burden with flexible response windows and fewer intrusive prompts.	Low
Avoid unintended positive or negative effects on mood	Design ambulatory assessment tools to minimize effects on mood by limiting app-based feedback on the mood itself but encourage continued completion of tools.	Moderate
Data security	Clearly communicate privacy policies and allow user control over data sharing.	Moderate
Participant support	Provide onboarding and support (eg, technical issues), ideally by someone with a trusted relationship to the participant.	Moderate
Attrition reduction and adherence improvement	Address barriers (stress, usability) and test onboarding strategies to improve retention.	Very low
Attrition and adherence reporting	All studies should systematically report these data and consider using a variety of measures to monitor adherence, for example, self-report versus app data.	High
Human contact	Consider the user preference that ambulatory assessment supplements—not replaces—meaningful interpersonal interaction.	Very low

a BD: bipolar disorder.

## Discussion

### Recommendations for Future Development—Mood Monitoring Interventions

#### Future Research Focus

Future research should definitively establish whether mood monitoring interventions have benefits for mood or other outcomes by themselves in people with BD and depression. These interventions may be most successful in asymptomatic individuals with good insight who are at risk of relapse, although this decreased relapse risk for asymptomatic low-risk individuals was only found in 1 RCT [33]. These should be evaluated with an appropriate control group that allows the effectiveness of mood monitoring alone to be assessed, rather than mood monitoring in addition to many other components, such as cognitive behavioral therapy. The vast majority of trials used mood monitoring as an outcome assessment in both arms, and so we were unable to investigate the effectiveness of mood monitoring alone in these studies. There is a tension in our results: in the qualitative work, many participants report that they value self-monitoring and that it is effective for them, while the quantitative studies do not show an effect in reducing symptom severity, but it is possible that the benefits of mood monitoring are more indirect through more sense-making and empowerment to take action and have hope of mood improvement and may be further exacerbated via the Rosenthal effect. Trials in mood monitoring assume that symptom reduction is the goal of self-management and alternative models may include a focus on functioning and quality of life despite symptoms.

#### Mechanism of Action

This qualitative research suggests effectiveness and provides clear mechanisms for relapse prevention. We demonstrate that users record their mood to then guide a wide range of self-management strategies. Mood monitoring allows users to improve their insight into their condition and assess their mood objectively. Some of the self-management strategies that followed improved insight included lifestyle adjustments to then prevent relapse. Many of these self-management strategies were highly personalized and iteratively developed over time to work optimally for the individual, as they increasingly gained insight into their disorder. Some of these strategies reported by participants included secondary prevention strategies: establishing a dialog with trusted individuals about their mood and how to manage it (eg, trusted individuals, friends or family), considering how to improve their sleep, doing more exercise at the right times, and thinking carefully about additional interventions to stop their mood from worsening, for example, contacting a health care professional. These strategies generally combine the expertise of the experience of the patient with the clinical professionalism of the clinician to provide a richer picture of the context and care required. Any form of integrated care planning with professionals, however, will depend on IT system integration, which carries further risks around data security; therefore, the benefits would need to be clearly demonstrated.

#### Personalization

Because these self-management techniques are so variable, it would be very difficult to incorporate them into a mood monitoring intervention. The protocol, however, could represent a framework that offers data to then allow the user to use this new information to then deploy their own self-management strategies in a highly personalized way. This kind of personalization and flexibility was important to both people with depression and BD, and the intervention could be constructed to support this. For example, the technology used by the intervention could be less personalized, but the way the data are used might be more highly personalized—for example, in basic mood monitoring data being used in nuanced and iterative ways for self-management. As the technology becomes more personalized, it is likely that many people will wish for some functions to be disabled, as they do not consider them appropriate for themselves at that time. For example, users wanted different amounts of control and autonomy over the collection of their behavioral and mood data, such as toggling off certain functions that were deemed intrusive. This would also help to alleviate data privacy concerns, which were common, but not shared by all participants.

#### Interface With Mental Health Services

We studied how these interventions might communicate with and be supported by mental health services and if this was desired by people with depression or BD. We proposed three different options: (1) self-management or monitoring without any involvement of mental health services, (2) mood monitoring occasionally shared with mental health services, and (3) clinician-directed monitoring. Many users with depression felt that sharing the data with their clinician was essential for the purpose of the ambulatory assessment, for example, providing accountability, setting goals, and using the data to have a detailed discussion with their health care professional around management strategies. Many people with BD, however, expressed skepticism about the help and support they would receive from mental health services and did not wish for the intervention to be integrated into their management by these services, instead using it in a personal sphere to self-manage their condition. Mood monitoring involving trusted individuals or family members was, however, frequently mentioned by users but rarely incorporated into the protocol itself.

#### Customizability

We thus advise that customizability be fundamental to future protocol development and incorporated using the recommendations made in this paper to maximize user engagement and successful uptake, and there are good methodological justifications for this [34,35]. Improving the “fit” of the intervention is likely to improve engagement and outcome [36]. People with depression or BD wished for the following types of personalization: the type of data collected, the data shared, the feedback the protocol offers, and notifications or wearables. Participants wished for an intuitive and easy-to-use passive data protocol that builds on their existing strengths with a high emphasis on personalization and customizability. Adapting and digitizing existing strengths-based models may improve user experience [37,38].

Incorporating these measures may improve adherence and attrition and potentially address some adverse events, such as increases in stress and how confronting the process feels. Future protocols should test additional therapeutic elements in the hope of managing these adverse events, which may present a significant barrier to future uptake. Furthermore, co-design and the use of responsible research and innovation techniques are both ways to de-risk innovations and interventions [39].

#### The Ideal Mood Monitoring Intervention

In our view, the ideal mood monitoring intervention would be based on a wide variety of passive data from smartphone sensors and other wearables (these measures are evolving and may include complex audio-visual data analysis [40]). It would have good performance, and users could trust that the mood prediction was accurate. It may be that passive data alone are not good enough to predict mood changes, and users may need to combine these data with an established mood measure to confirm mood change if the passive data detect an early shift. The intervention would be customizable in the sense that users could toggle data on and off if one particular aspect was deemed intrusive. The data would be stored in a secure way, and data sharing is consented to by the user in advance. The intervention would not have any technological problems—these were frequently described by participants as a barrier to use. Passive data methods, however, are not always low burden, as some individuals found passive wearables burdensome (eg, wearing an uncomfortable smartwatch overnight that may interfere with sleep [17]). Even with the simplest active ambulatory assessment measures, such as single-item mood reporting, many individuals found these intrusive and burdensome, particularly when depressed. Ambulatory assessment protocols should therefore allow the personalization of data collection methods and frequency to account for differing perceptions of burden [41].

The actual intervention could be relatively simple—providing occasional and clear feedback when a user’s mood is deteriorating—but these data could be used in a far more nuanced and personalized way. For example, this can then allow the user to implement self-management strategies that they may have already developed and hopefully prevent relapse, for example, via a well-being, recovery, or crisis plan. The intervention could remind the user of their crisis plan, and the user could preselect things they wish to be reminded of when their mood deteriorates. The intervention could serve both to detect early warning signs of mood deterioration but also to retrospectively assess mood over the long term to allow for appraisal of their self-management strategies and provide hope that dips can be recovered with appropriate support. These data could also be shared to guide decision-making, for example, medication choices and assessing effectiveness with objective data. These self-management strategies may or may not include involvement with mental health services, and any wish for involvement should not be assumed.

Particularly for people with depression, the intervention should aim to incorporate therapeutic elements to address adverse events—in particular the potential for subjective worsening of mood and how confronting the protocol can be. Any feedback the app offers should be co-designed with people with lived experience of BD or depression, as we report some feedback mechanisms via protocols that participants felt were patronizing and were detrimental to use. Participants wished for use to feel positive and to build on their strengths—while acknowledging that this may not always be possible. This may demonstrate a preference for selective reflection, such as that used in other therapeutic techniques, for example, motivational interviewing—where there is a slight bias toward adopting healthier habits. Medication reminders were perceived positively. Some protocols may require some form of onboarding or preparation to use—this is to consider purpose and apply some of the potential uses of a mood monitoring tool to someone’s life in order for them to maximize its benefits. This could be with a clinician or with a digital navigator—someone who knows and understands both the technology but also the health challenges that people may have through their disability or ill-health [42,43]. The app would not replace clinician time but be used as an additional tool for people to self-manage their mental illness, or improve the efficiency of time spent with a clinician. Insight gained during tracking is a potential tool for shared decision-making and may therefore contribute toward patient activation.

### Recommendations for Ambulatory Assessment in Research

#### Performance

The performance of these protocols was unclear, particularly when assessing passive measures of mood. The performance of active ambulatory assessment was good (correlation coefficients: moderate or strong strength), but this was often using already validated measures of mood, administered more frequently. Performance is a key issue for people with mood disorders, as it signals the degree of trust (eg, and thus adherence or attrition) they should place in any feedback that is provided, which in turn can cause them to adjust the management of their illness.

#### Standardization

This review highlights the need for possible standardization in ambulatory assessment methodologies (eg, in the frequency, duration, devices used, interventions delivered, association or validation) to support the implementation of these behavioral assessment measures. In depression, 423 different smartphone-based passive ambulatory assessment performance measures were extracted, but few overlapped across studies, and many lacked validation or comparative measures. Current performance, particularly with passive ambulatory assessment measures, demonstrates large methodological variation and inconsistent reporting. The wide range of ambulatory assessment protocols used across studies and the differences in reporting metrics severely limit the ability to compare performance outcomes. Standardized reporting frameworks may address some of these issues, ensuring findings can be reliably replicated and robustly compared.

It might not be plausible, however, to standardize ambulatory assessment protocols relatively quickly, as the depression measurement tools we identified here varied significantly—from established questionnaires to some simple question or questions and from a full scale to a couple of core items or even a single item. To compare the results from different studies with various depression measures, we propose the following approaches.

One method is to use standardized quantities such as the standardized mean difference or Cohen *d*, which were derived from different studies [44]. The disadvantages of this, however, are that scale-free standardized scores lack contextual meaning, are sensitive to variability, and have arbitrary interpretations [45,46]. Another possible method is to convert the scores between different depression questionnaires [47-49]. This method also has its limitations, and it ignores differences in the questionnaire’s content validity and structure. For example, the 17-item Hamilton Depression Rating Scale and the 9-item Patient Health Questionnaire measure different components of depression [50,51], and the underlying constructs of many different depression questionnaires differ slightly [52]. Another limitation is that the conversion does not reliably preserve severity‐category alignment (eg, mild, moderate, severe), and this can lead to mismatches in the clinical interpretation [53]. Considering the above methodological limitations and the widespread use of certain depression measures, we suggest using established depression measures that have sound psychometric characteristics in ambulatory assessment, and this must be guided by clinical need.

#### The Ideal Ambulatory Assessment Research Protocol

In our view, the ideal ambulatory assessment research protocol would be digital and rely principally on passive data but may need to confirm or validate any mood shift detected using passive data with a validated self-report measure, for example, the 9-item Patient Health Questionnaire. This is because passive data collection can only detect observable changes in behavior or physiological function; it cannot measure the subjective experience of mood that self-rated measures reveal. Digital methods allow for more customizability and personalization and are perceived as being easier to use. Any self-report measures should aim to be triggered at a time that is convenient for the participant, for example, self-selected in advance with an option to delay the assessment within a reasonable timeframe. This is because passive approaches are favored by many participants and may address concerns that active ambulatory assessment can be stressful and perceived as a burden, and active engagement can be a distressing reminder of one’s mental illness. It also overcomes issues with recall bias and helps participants with mood disorders who struggle to fill in assessments when they are more severely unwell, for example, due to memory, concentration, or motivation issues.

Ideally, the ambulatory assessment would not exert any effects on mood, which could obfuscate or confound the results in intervention studies. This, however, may be unrealistic (eg, Hawthorne effect), and it may be possible that the frequent assessment will either deteriorate or improve mood, especially if there is any feedback on the mood itself, and this information is used by the participant, consciously or unconsciously, to make adaptations in their life [54]. There would be notifications to complete the ambulatory assessment, but the user would have some control over their frequency. The ambulatory assessment should be carefully tested and free of major technological issues, which are a frequent barrier to use. Some participants reported that frequent crashes and syncing problems with wearables were their reasons for dropping out, increased the perceived burden of using the app, and undermined trust in the feedback provided by the app. Participants may require support prior to using the ambulatory assessment, for example, demonstrating use, what can be controlled by them (eg, notifications), what cannot be controlled. This is likely to be most effective when this initial support comes from a known or trusted person rather than a general tutorial or automated system. Again, the use of the ambulatory assessment protocol should not completely replace human contact, and participants still valued this. The initial assessment may benefit from being in person or performed by someone who the participant already has a trusting relationship with. This may also improve attrition or adherence, although this hypothesis requires further empirical evaluation. Researchers should be able to address data security concerns that are common and of concern to people with mood disorders.

Some of our analyses were limited by a paucity of reporting of performance measures, particularly those in forms that would be easily comparable between studies. Future research should report the performance of ambulatory assessment protocols in standardized ways in accordance with other areas of scientific research [55].

### Strengths and Limitations of the Work

This paper builds on previous work to triangulate the findings from a wealth of quantitative and qualitative data exploring optimum development from a wide variety of perspectives. The recommendations themselves are drawn by the team that performed the systematic review bringing together psychiatrists, methodologists, and service users who would necessarily agree on their nature or importance. The findings therefore catalytic validity. The literature itself is drawn from a range of Western countries and not from low- and middle-income countries, which is an important gap in the research. The evidence was not put to an independent external interdisciplinary group of experts, and we did not use any consensus-drawing methodology such as Delphi. Therefore, the recommendations are not definitive but interim and suitable for a field where technological innovation is likely to quickly progress as artificial intelligence, wearables, bots, and avatars are developed and used to monitor, measure, and manage mood.

### Conclusions

We assess ambulatory assessment protocols and mood monitoring interventions and provide clear evidence-based recommendations for future development. Future iterations of ambulatory assessment protocols and mood monitoring interventions should consider these findings and how they might incorporate some of these themes into development. Crucially, the requirements and recommendations are different depending on the purpose, for example, ambulatory assessment for research or digital mood monitoring intervention. There are, however, overlapping themes, such as personalization and performance. These recommendations have been formulated closely with people with lived experience, by reviewing qualitative research and having the results checked for salience and coherence by individuals with lived experience and clinicians. Future ambulatory assessment protocols must balance personalization, performance, and safety if they are to fulfill their substantial promise in both research and clinical practice. None of these measures are yet robust enough to replace current outcomes, but they can add additional granularity and confirmation (since they are free of recall bias and collected in the moment). There remains great potential for these measures if the detailed challenges can be overcome.

## Supplementary material

10.2196/79501 Multimedia Appendix 1 Characteristics of included qualitative and quantitative studies in people with bipolar disorder and depression.

## Abbreviations

BD: bipolar disorder
EMA: ecological momentary assessment
GRADE: Grading of Recommendations Assessment, Development and Evaluation
RCT: randomized controlled trial

## References

[1] Shiffman S, Stone AA, Hufford MR (2008). Ecological momentary assessment. Annu Rev Clin Psychol.

[2] het Rot MA, Hogenelst K, Schoevers RA (2012). Mood disorders in everyday life: a systematic review of experience sampling and ecological momentary assessment studies. Clin Psychol Rev.

[3] Gromatsky M, Sullivan SR, Spears AP (2020). Ecological momentary assessment (EMA) of mental health outcomes in veterans and servicemembers: a scoping review. Psychiatry Res.

[4] Armey MF, Schatten HT, Haradhvala N, Miller IW (2015). Ecological momentary assessment (EMA) of depression-related phenomena. Curr Opin Psychol.

[5] De Angel V, Lewis S, White K (2022). Digital health tools for the passive monitoring of depression: a systematic review of methods. NPJ Digit Med.

[6] Bladon S, Eisner E, Bucci S (2025). A systematic review of passive data for remote monitoring in psychosis and schizophrenia. NPJ Digit Med.

[7] Dubad M, Winsper C, Meyer C, Livanou M, Marwaha S (2018). A systematic review of the psychometric properties, usability and clinical impacts of mobile mood-monitoring applications in young people. Psychol Med.

[8] de Vries LP, Baselmans BML, Bartels M (2021). Smartphone-based ecological momentary assessment of well-being: a systematic review and recommendations for future studies. J Happiness Stud.

[9] Bos FM, van der Krieke L, Wichers M, Bruggeman R, Snippe E (2023). Ecological momentary assessment as a clinical tool in psychiatry: promises, pitfalls, and possibilities. Tijdschr Psychiatr.

[10] Piot M, Mestdagh M, Riese H (2022). Practitioner and researcher perspectives on the utility of ecological momentary assessment in mental health care: a survey study. Internet Interv.

[11] Onnela JP, Rauch SL (2016). Harnessing smartphone-based digital phenotyping to enhance behavioral and mental health. Neuropsychopharmacology.

[12] Ebner-Priemer UW, Mühlbauer E, Neubauer AB (2020). Digital phenotyping: towards replicable findings with comprehensive assessments and integrative models in bipolar disorders. Int J Bipolar Disord.

[13] (2025). Advancing the use of sensor-based digital health technologies (sDHTs) for mental health research and clinical practice. https://wellcomeopenresearch-files.f1000.com/posters/docs/wellcomeopenres-363664.pdf?_ga=undefined.

[14] Astill Wright L, Moore M, Reeves S, Vallejos EP, Morriss R (2025). Improving the utility, safety, and ethical use of a passive mood-tracking app for people with bipolar disorder using coproduction: qualitative focus group study. JMIR Form Res.

[15] White KM, Dawe-Lane E, Siddi S (2023). Understanding the subjective experience of long-term remote measurement technology use for symptom tracking in people with depression: multisite longitudinal qualitative analysis. JMIR Hum Factors.

[16] Astill Wright L, Majid M, Moore M (2025). The user experience of ambulatory assessment and mood monitoring in bipolar disorder: systematic review and meta-synthesis of qualitative studies. J Med Internet Res.

[17] Astill Wright L, Majid M, Shajan G (2025). The user experience of ambulatory assessment and mood monitoring in depression: a systematic review & meta-synthesis. NPJ Digit Med.

[18] Schneider S, Junghaenel DU, Smyth JM, Fred Wen CK, Stone AA (2024). Just-in-time adaptive ecological momentary assessment (JITA-EMA). Behav Res Methods.

[19] Atkins D, Best D, Briss PA (2004). Grading quality of evidence and strength of recommendations. BMJ.

[20] Bos FM, Snippe E, Bruggeman R, Doornbos B, Wichers M, van der Krieke L (2020). Recommendations for the use of long-term experience sampling in bipolar disorder care: a qualitative study of patient and clinician experiences. Int J Bipolar Disord.

[21] Van der Watt ASJ, Roos T, Beyer C, Seedat S (2018). Participants’ perspectives of weekly telephonic mood monitoring in South Africa: a feasibility study. Pilot Feasibility Stud.

[22] Bos FM, Snippe E, Bruggeman R, Wichers M, van der Krieke L (2019). Insights of patients and clinicians on the promise of the experience sampling method for psychiatric care. Psychiatr Serv.

[23] Perez Arribas I, Goodwin GM, Geddes JR, Lyons T, Saunders KEA (2018). A signature-based machine learning model for distinguishing bipolar disorder and borderline personality disorder. Transl Psychiatry.

[24] Benedyk A, Moldavski A, Reichert M (2023). Initial response to the COVID-19 pandemic on real-life well-being, social contact and roaming behavior in patients with schizophrenia, major depression and healthy controls: a longitudinal ecological momentary assessment study. Eur Neuropsychopharmacol.

[25] Emden D, Goltermann J, Dannlowski U, Hahn T, Opel N (2021). Technical feasibility and adherence of the Remote Monitoring Application in Psychiatry (ReMAP) for the assessment of affective symptoms. J Affect Disord.

[26] Simblett S, Matcham F, Curtis H (2020). Patients’ measurement priorities for remote measurement technologies to aid chronic health conditions: qualitative analysis. JMIR Mhealth Uhealth.

[27] Bonilla-Escribano P, Ramírez D, Baca-García E, Courtet P, Artés-Rodríguez A, López-Castromán J (2023). Multidimensional variability in ecological assessments predicts two clusters of suicidal patients. Sci Rep.

[28] Simblett S, Dawe-Lane E, Gilpin G (2024). Data visualization preferences in remote measurement technology for individuals living with depression, epilepsy, and multiple sclerosis: qualitative study. J Med Internet Res.

[29] Sharma AN, Barron-Millar E, Gaskell M (2022). Technology matters: collaboratively augmenting longitudinal monitoring (C.A.L.M) in bipolar disorder—co-design, co-production and evaluation of the alpha prototype app. Child Adolesc Ment Health.

[30] Funkhouser CJ, Trivedi E, Li LY (2024). Detecting adolescent depression through passive monitoring of linguistic markers in smartphone communication. J Child Psychol Psychiatry.

[31] Webb CA, Tierney AO, Brown HA, Forbes EE, Pizzagalli DA, Ren B (2022). Spontaneous thought characteristics are differentially related to heightened negative affect vs. blunted positive affect in adolescents: an experience sampling study. JCPP Adv.

[32] Hetrick SE, Robinson J, Burge E (2018). Youth codesign of a mobile phone app to facilitate self-monitoring and management of mood symptoms in young people with major depression, suicidal ideation, and self-harm. JMIR Ment Health.

[33] Goulding EH, Dopke CA, Rossom R, Jonathan G, Mohr D, Kwasny MJ (2023). Effects of a smartphone-based self-management intervention for individuals with bipolar disorder on relapse, symptom burden, and quality of life: a randomized clinical trial. JAMA Psychiatry.

[34] Myin-Germeys I, Kuppens P (2022). The Open Handbook of Experience Sampling Methodology: A Step-by-Step Guide to Designing, Conducting, and Analyzing ESM Studies.

[35] Silvia PJ, Cotter KN (2021). Researching Daily Life: A Guide to Experience Sampling and Daily Diary Methods.

[36] Bertolino B (2012). The Therapist’s Notebook on Positive Psychology: Activities, Exercises, and Handouts.

[37] Niemiec RM, Pearce R (2021). The practice of character strengths: unifying definitions, principles, and exploration of what’s soaring, emerging, and ripe with potential in science and in practice. Front Psychol.

[38] Schrank B, Bird V, Rudnick A, Slade M (2012). Determinants, self-management strategies and interventions for hope in people with mental disorders: systematic search and narrative review. Soc Sci Med.

[39] Ito-Jaeger S, Lane G, Dowthwaite L (2023). TrustScapes: a visualisation tool to capture stakeholders’ concerns and recommendations about data protection, algorithmic bias, and online safety. Int J Qual Methods.

[40] (2025). TrueBlue clinical study—investigating the use of a mobile phone app trueblue for monitoring depression and anxiety. Health Research Authority.

[41] Craven MP, Goodwin R, Rawsthorne M (2019). Try to see it my way: exploring the co-design of visual presentations of wellbeing through a workshop process. Perspect Public Health.

[42] Perret S, Alon N, Carpenter-Song E (2023). Standardising the role of a digital navigator in behavioural health: a systematic review. Lancet Digit Health.

[43] Wisniewski H, Gorrindo T, Rauseo-Ricupero N, Hilty D, Torous J (2020). The role of digital navigators in promoting clinical care and technology integration into practice. Digit Biomark.

[44] Lipsey MW, Wilson D (2000). Practical Meta-Analysis.

[45] Cohen J (1988). Statistical Power Analysis for the Behavioral Sciences.

[46] Cumming G (2013). Cohen’s d needs to be readily interpretable: comment on Shieh (2013). Behav Res Methods.

[47] Rush AJ, Trivedi MH, Ibrahim HM (2003). The 16-item quick inventory of depressive symptomatology (QIDS), clinician rating (QIDS-C), and self-report (QIDS-SR): a psychometric evaluation in patients with chronic major depression. Biol Psychiatry.

[48] Hawley CJ, Gale TM, Smith VR, Sen P (1998). Depression rating scales can be related to each other by simple equations. Int J Psychiatry Clin Pract.

[49] Carmody TJ, Rush AJ, Bernstein I (2006). The Montgomery Asberg and the Hamilton ratings of depression: a comparison of measures. Eur Neuropsychopharmacol.

[50] Guo B, Kaylor-Hughes C, Garland A (2017). Factor structure and longitudinal measurement invariance of PHQ-9 for specialist mental health care patients with persistent major depressive disorder: exploratory structural equation modelling. J Affect Disord.

[51] Nixon N, Guo B, Garland A, Kaylor-Hughes C, Nixon E, Morriss R (2020). The bi-factor structure of the 17-item Hamilton Depression Rating Scale in persistent major depression; dimensional measurement of outcome. PLOS One.

[52] Rush AJ, Bernstein IH, Trivedi MH (2006). An evaluation of the quick inventory of depressive symptomatology and the Hamilton Rating Scale for depression: a sequenced treatment alternatives to relieve depression trial report. Biol Psychiatry.

[53] Cameron IM, Crawford JR, Cardy AH (2013). Psychometric properties of the Quick Inventory of Depressive Symptomatology (QIDS-SR) in UK primary care. J Psychiatr Res.

[54] Brown S, Ploeger C, Guo B (2024). When a test is more than just a test: findings from patient interviews and survey in the trial of a technology to measure antidepressant medication response (the PReDicT Trial). Compr Psychiatry.

[55] Cohen JF, Korevaar DA, Altman DG (2016). STARD 2015 guidelines for reporting diagnostic accuracy studies: explanation and elaboration. BMJ Open.

