# Supplementary material for: Recommendations for Research and Clinical Implementation of Ambulatory Assessment, Mood Monitoring, Digital Phenotyping, and Remote Measurement Technology in Mood Disorders: Synthesis of Systematic Review Findings
Source: JMIR Ment Health. 2026 Jun 2;13:e79501. doi: 10.2196/79501 (PMC13229395; doi:10.2196/79501)
Supplement: Multimedia Appendix 1 [file mental-v13-e79501-s001.docx]

| **Supplementary Table 1: Characteristics of included qualitative and quantitative studies in people with bipolar disorder and depression** | | | | | | | | | | | |
| --- | --- | --- | --- | --- | --- | --- | --- | --- | --- | --- | --- |
| **Characteristics of included qualitative studies in people with bipolar disorder** | | | | | | | | | | | |
| **Study** | **Country** | **Sample** | **n** | **Mean age in years for bipolar disorder (SD)** | **% female** | **Intervention** | **Setting** | **Active vs passive ambulatory assessment** | **EMA or Mood Monitoring** | **Ambulatory assessment /mood tracking  procedure** | **Ambulatory assessment duration** |
| Bos et al 2019 | Netherlands | Depression: 45%, Bipolar Disorder: 32%, Anxiety disorder: 18%, Psychosis: 14%, Eating disorder: 5%, Autism spectrum disorder: 5%, Unknown: 5% | Patients: 22, Clinicians: 21 | 20-35 years, n = 6, 36-50 years, n = 7, 51-65, n = 7, 66 or older, n = 2 | 64 | Hypothetical mood monitoring app | Mixed sample – participants recruited via secondary care outpatients and mental health institutions. | Active | Mood monitoring | Hypothetical app where individuals can record their moods, experiences, behaviors, contexts, and thoughts multiple times per day on their smart phones. | Hypothetical use |
| Bos et al 2020 | Netherlands | Bipolar 1: 45%, Bipolar 2: 55% | Bipolar disorder: 18, Clinicians: 6 | 20-35 years: n = 9, 36-50 years: n = 8, 51-65 years: n = 3 | 80 | 5x EMA smartphone assessments daily - 29 items assessing monetary mood, symptoms, sleep and activities. Weekly ASRM, QIDS-SR-16 delivered via RoQua platform. | Tertiary care - mood disorders service | Active | EMA | 5x EMA smartphone assessments daily - 29 items assessing monetary mood, symptoms, sleep and activities. Weekly ASRM, QIDS-SR-16 delivered via RoQua platform. | 4 months (range: 16-32 weeks) |
| Geerling et al 2021 | Netherlands | Not reported | Bipolar disorder: 11, clinicians: 6 | 16-24 years: 1, 25-40: 4, 41-55: 5, 56-70: 3 | 53.8 | NIMH Life Chart Methodology - prospective — daily mood self-rating | Mixed sample – participants recruited via secondary care outpatients and advocacy groups | Active | Mood monitoring | NIMH Life Chart Methodology - prospective — daily mood self-rating | Variable depending on protocol |
| Jonathan et al 2021 2 | USA | Not reported | 11 | 36 (14) | 63.6 | Livewell - smartphone based self management intervention | Secondary care – 1 previous mood episode in the past year and current care by psychiatrist/nurse practitioner | Active | Mood monitoring | Smartphone based self-management intervention - daily and weekly check-ins for weeks 1-16. Daily - adherence, sleep, duration, routine, wellness levels. Weekly - symptom severity scoring for all individual DSM-IV mood symptoms. | 8w |
| Jonathan et al 2021 | USA | Not reported | 12 | 38 (14) | 66 | Livewell - smartphone based self management intervention | Secondary care | Active | Mood monitoring | 1. Simple smartphone app for self-monitoring of behavioural targets. 2.  LiveWell: Smartphone based self management intervention - daily and weekly check-ins for weeks 1-16. Daily - adherence, sleep, duration, routine, wellness levels. Weekly - symptom severity scoring for all individual DSM-IV mood symptoms. | Simple smartphone app: 12 weeks. LiveWell: 8 weeks. |
| Jonathan et al 2024 | USA | Bipolar 1: 100% | 17 | 45 (12) | 53 | Livewell - smartphone based self management intervention | Secondary care | Active | Mood monitoring | Smartphone based self-management intervention - daily and weekly check-ins for weeks 1-16. Daily - adherence, sleep, duration, routine, wellness levels. Weekly - symptom severity scoring for all individual DSM-IV mood symptoms. | 48 weeks |
| Gordon-Smith et al 2023 | UK | Bipolar 1: 56.1%, Bipolar 2: 39.0%, Schizoaffective disorder - Bipolar type: 1.4%, Bipolar Disorder not otherwise specified: 3.6% | 362 | median: 54.0 (IQR: 17) | 67.4 | True Colours | Mixed sample – participants recruited via secondary care/advertising | Active | Mood monitoring | True Colours - ASRM/QIDS-SR16 delivered via weekly SMS/email | 28 months |
| Morton et al 2019 | Canada | Bipolar 1: 55%, Bipolar 2: 37%, Bipolar 2 rapid cycling: 2.3%, Bipolar Disorder not otherwise specified: 4.7% | 43 | Modal age range: 45-54 | 69 | Web-based version of a BD-specific QoL self-report measure - the QoL tool | Mixed sample – participants recruited via tertiary care/advertising | Active | Mood monitoring | Web-based version of a BD-specific QoL self-report measure - weekly | Not reported |
| Morton et al 2022 | Canada | Bipolar 1: 34.9%, Bipolar 2: 51.9%, Other/no formal diagnosis: 13.2% | 919 | 36.9 | 77.9 | Open enquiry into mood monitoring/tracking applications | Mixed sample – participants recruited via tertiary care/advertising | Active | Mood monitoring | Various mood/sleep monitoring/tracking applications: Daylio, Bipolar eMoods Tracker, iMood Journal, Moodpath, Calm, FitBit, Sleep Cycle, Samsung Health, Headspace. | Self-report previous use |
| Murnane et al 2015 | USA | Bipolar 1: 27%, Bipolar 2: 41%, Bipolar Disorder not otherwise specified: 17%, Cyclothymia: 2%, Not known: 9%, Declined: 4% (exact percentages not given) | 552 | Under 18: 1%, 18-24: 8%, 25-34: 24%, 35-44: 28%, 45-54: 23%, 55-64: 14%, 65-74: 2% (exact percentages not given) | 83 | Open enquiry into behaviour/health tracking practices | Mixed sample – participants recruited online | Active | Mood monitoring | Various mood/sleep monitoring/tracking applications: Charting software, digital notebooks, spreadsheets, smartphone applications, online mood trackers. | Self-report previous use |
| Murray et al 2011 | Canada | Bipolar 1: 78.1%, Bipolar 2: 21.9% | 33 | 41.1 (13.3) | 63 | Open enquiry into behaviour/health tracking practices | Mixed sample – participants recruited via advertising in person and online | Active | Mood monitoring | Various analogue mood/sleep monitoring/tracking strategies | Self-report previous use |
| Nicholas et al 2017 | Australia | Not reported | 89 | 24.4 (3.9) | 87 | Open enquiry into mood monitoring/tracking applications | Mixed sample – participants recruited online | 16 different apps used for mood monitoring | Mood monitoring | Various mood/sleep monitoring/tracking applications | Self-report previous use |
| Rusch et al 2022 | USA | Bipolar 1: 83.3%, Bipolar 2: 11.1% | total: 91, qualitative interviews: 18 | 49.2 (13.7) | 66.7 | Life Goals app | Existing cohort | Active | Mood monitoring | Life Goals app: mood monitoring symptoms of anxiety, depression and mania using the PHQ-9, GAD-7, Internal State Scale | 6 months |
| Saunders et al 2017 | UK | Bipolar 1: 66.6%, Bipolar 2: 33.3% | 21 | 44.38 | 66.7 | True Colours | Mixed sample – participants recruited via secondary care/advertising | Active & passive ambulatory assessment | Mood monitoring & EMA | True Colours - ASRM/QIDS-SR16 delivered via weekly SMS/email. Daily mood monitoring via smartphone app and wearables. One week of 10x daily EMA and continuous use of wearables. | 3 months |
| Sharma et al 2022 | UK | Not reported | 13 | Range: 14.5 to 24.2 | 92.3 | Collaboratively augmenting longitudinal monitoring (C.A.L.M) in Bipolar Disorder | Not reported | Active | Mood monitoring | CALM in Bipolar Disorder: mood monitoring | 3 months |
| Stern & Sin 2012 | UK | Not reported | 23 | Not reported | Not reported | Structured Group Psychosocial Intervention | Secondary care | Active | Mood monitoring | Structured Group Psychosocial Intervention - incorporating mood monitoring | 3 months |
| Suto et al 2009 | Canada | Bipolar 1: 78.1%, Bipolar 2: 21.9% | 32 | 41.1 (13.3) | 63 | Open enquiry into behaviour/health tracking practices | Mixed sample – participants recruited via advertising in person and online | Active | Mood monitoring | Various self management strategies including monitoring | Self-report previous use |
| Todd et al 2012 | UK | Bipolar 1: 66.6%, Bipolar 2: 33.3% | 12 | 42 | 41.6 | Living With Bipolar | Mixed sample – participants recruited via advertising in person and online | Active | Mood monitoring | Living With Bipolar - web-based self-management intervention | Exploratory study without testing of the intervention |
| van Bendegem et al 2014 | Netherlands | Bipolar 1: 92.9%, Bipolar 2: 7.1% | 14 | 41.5 | 50 | NIMH Life Chart Methodology - prospective — daily mood self-rating | Secondary care | Active | Mood monitoring | NIMH Life Chart Methodology - prospective — daily mood self-rating | 2 months - 3 years |
| Van der Watt et al 2018 | Netherlands | Bipolar Disorder: 35.1%, Depression: 56.8%, Mood and anxiety disorder: 2.7%, Other affective disorder: 5.4% | 37 | 35.76 (10.8) | 89.2 | Weekly telephone mood monitoring | Secondary care | Active | Mood monitoring | Weekly telephone mood monitoring | 6.5 months |
| **Characteristics of included qualitative studies in people with depression** | | | | | | | | | | | |
| Bos et al 2019 | Netherlands | Depression: 45%, Bipolar Disorder: 32%, Anxiety disorder: 18%, Psychosis: 14%, Eating disorder: 5%, Autusm spectrum disorder: 5%, Unknown: 5% | Patients: 22, Clinicians: 21 | 20-35 years, n = 6, 36-50 years, n = 7, 51-65, n = 7, 66 or older, n = 2 | 64 | Open enquiry into mood monitoring/tracking applications | Mixed sample – participants recruited via secondary care outpatients and mental health institutions. | Active | Mood monitoring | Hypothetical app where individuals can record their moods, experiences, behaviors, contexts, and thoughts multiple times per day on their smart phones. | Hypothetical use |
| de Angel et al 2022 | UK | Adults with depression: 16, Clinicians: 6 | 22 | 44.6 (13.3) | 82 | RADAR-base system | Primary care - IAPT/NHS Talking therapies | Passive | Mood monitoring | Hypothetical discussion around RADAR-base passive monitoring | Hypothetical discussion around RADAR-base passive monitoring |
| de Angel et al 2023 | UK | Adults with depression | 66 | 34.6 (11.1) | 61 | RADAR-base system | Primary care - IAPT/NHS Talking therapies | Active & passive | Mood monitoring | RADAR-base system PHQ-8, RSES, speech task – weekly. Heartrate, step-count, GPS location, acceleration, light levels, phone interaction, nearby Bluetooth device detection, battery level, weather, sleep, app usage metrics – continuous via Fitbit/RADAR-base app. | 7 months |
| Drake et al 2012 | UK | Adults with depression | 16 | Completers: 38.9 (12.6), Dropouts: 32.8 (15.3) | 75 | Moodscope | Primary care | Active | Mood monitoring | PHQ-9, GAD-7 - weekly. Moodscape self-rated mood - selecting which of each 20 interactive mood-adjective playing cards describes current mood. | 3 months |
| Folkersma et al 2021 | UK | Adults with depression | 20 | 18-30: 50%, 31-45: 20%, 46-65: 30% | 35 | ZELF-I | Secondary care | Active | Mood monitoring | ZELF-I: 5 brief questionnaires per day for 28 days | 1 month |
| Hetrick et al 2018 | Australia | Young people with depression: 11, Clinicians: 16 | 27 | 21.4 | 73 | Customisable mood monitoring | Mixed sample – participants recruited via secondary care/advertising | Active | Mood monitoring | User centred development of mood monitoring app | Minimal use - Hypothetical discussion around development |
| Incecik et al 2020 | UK | Adults with treatment resistant depression | 21 | 41.2 (15.2) | 38 | True Colours | Mixed sample – participants recruited via secondary care/advertising | Active | Mood monitoring | True Colours - ASRM/QIDS-SR16 delivered via weekly SMS/email | 1 year |
| Meng et al 2018 | USA | Adults with depression: 12, Clinicians: 9 | 21 | Adults with depression: 21 (1.22), Clinicians: 42 (5.83) | Adults with depression: 58, Clinicians: 89 | iSee - conceptual prototype | Primary care | Passive | Mood monitoring | iSee - conceptual prototype using passive ambulatory assessment | Minimal use - Hypothetical discussion around development |
| Patoz et al 2021 | France | Adults with depression: 24, Clinicians: 26 | 50 | Adults with depression: 51.5 (15.5), Clinicians: 45.5 (12.2) | Adults with depression: 54.1, Clinicians: 50.0 | Hypothetical depression app | Secondary care | Active | Mood monitoring | Hypothetical depression app | Minimal use - Hypothetical discussion around development |
| Simblett et al 2020 | UK | Adults with depression: 8, Epilepsy: 7, Multiple sclerosis: 9 | 24 | Adults with depression: 51.9 (9.4), Epilepsy: 44.4 (15.8), Multiple sclerosis: 43.4 (9.5) | Adults with depression: 63, Epilepsy: 71, Multiple sclerosis: 67 | RADAR-base system | Mixed sample – participants recruited via advertising | Active & passive | Mood monitoring | RADAR-base system PHQ-8, RSES, speech task – weekly. Heartrate, step-count, GPS location, acceleration, light levels, phone interaction, nearby Bluetooth device detection, battery level, weather, sleep, app usage metrics – continuous via Fitbit/RADAR-base app. | Minimal use - Hypothetical discussion around development |
| Simblett et al 2024 | UK | Adults with depression: 17, Epilepsy: 11, Multiple Sclerosis: 17 (Only data on depression included here) | 17 | 55 (13) | 83 | RADAR-base system | Mixed sample – participants recruited via advertising | Active & passive | Mood monitoring | RADAR-base system PHQ-8, RSES, speech task – weekly. Heartrate, step-count, GPS location, acceleration, light levels, phone interaction, nearby Bluetooth device detection, battery level, weather, sleep, app usage metrics – continuous via Fitbit/RADAR-base app. | Minimal use - Hypothetical discussion around development |
| Thomson et al 2024 | Austrailia | Adults with depression | 10 | 26 (Q1 - Q3: 20 - 50) | 70 | Co-HIVE | Secondary care | Active & passive | Mood monitoring | Co-HIVE system: smartphone application (Mentegram - administering PHQ-9, HDRS, BDI-II, DASS-21, C-SSRS) and smartwatch device for remote health monitoring (Fitbit Charge 5 - heart rate, stepcount, sleep), telehealth appointments (HealthDirect) for virtual check-ins/health coaching | 10-12 weeks |
| Van Tiem et al 2021 | USA | Adults with depression | 21 | 48 (range: 28-72) | 19 | Daily test messaging intervention | Veterans Affairs | Active | Mood monitoring | Daily test messaging intervention including mood monitoring - daily text message exchanges with an automated system and weekly secure messages with visual representations of the participants messages. Linked to electronic health record and can be viewed by clinician. | 12w |
| White et al 2023 | UK, Spain, Netherlands | Adults with depression | 99 | Time point - 3 months: 44.6 (12.1), 12 months: 49.4 (13.5), 24 months: 51.9 (15.0) | Time point - 3 months: 75, 12 months: 76, 24 months: 69 | RADAR-base system | Mixed sample – participants recruited via secondary care/advertising | Active & passive | Mood monitoring | RADAR-base system PHQ-8, RSES, speech task – weekly. Heartrate, step-count, GPS location, acceleration, light levels, phone interaction, nearby Bluetooth device detection, battery level, weather, sleep, app usage metrics – continuous via Fitbit/RADAR-base app. | 2 years |
| **Included randomised controlled trials in people with depression** | | | | | | | | | | |  |
| **Study** | **Country** | **n** | **Mean age in years (SD)** | **% female** | **Intervention** | **Comparator** | **Setting** | **Ambulatory assessment/mood tracking procedure** | **Ambulatory assessment duration** | **Established Mood Outcome** |  |
| Aikens et al 2022 | USA | 204 | 49 | 81 | Automated Interactive Voice Response telephone calls | Enhanced usual care - usual care plus printed self-management material at baseline and assigned family/friend to discuss this with weekly | Primary care | Automated Interactive Voice Response telephone calls assessing symptom severity - PHQ-9 and antidepressant adherence. | 12 months | PHQ-9 |  |
| van Genugten et al. 2021 | Netherlands | 943 | 37.3 (13.2) | 66 | bCBT: psychoeducation, behavioural activation, cognitive restructuring, relapse prevention | TAU | Primary and secondary care | Daily self-monitoring of mood state via VAS, cognitions, activities, social interaction, and sleep via a smartphone app. Ambulatory assessment protocol varied over course of the study, one some days 3x daily mood ratings. | 12w | MINI - baseline, PHQ-9 - 3 months |  |
| Arean et al 2016 | USA | 1110 | 18-30: 584 (53.6%), 31-40: 243 (22.4%), 41-50: 151 (13.9%), 51-60: 84 (7.7%), 61-70: 20 (1.8%), 71+: 6 (0.6%) | 78.4 | Intervention arm 1: iPST - an app based on problem solving therapy, Intervention arm 2: Project Evo - a therapeutic video game meant to improve cognitive skills associated with depression, Intervention arm 3: Health Tips - an app that provides information about strategies to improve mood. | As per intervention column | Mixed sample – participants recruited via multiple settings. | Active ambulatory assessment: PHQ-9, SDS , Global Impression of Change Scale, 3 question sleep assessment, Mental Health Services Used - weekly. PHQ-2 - daily. Passive ambulatory assessment - time of call/SMS, call duration, SMS length, activity type, distance travelled. | 12w | PHQ-9, SDS, GAD-7, AUDIT-C, IMPACT Mania & Psychosis — baseline |  |
| Pratap et al 2018 | USA | 1083 | 18-30: 456 (43.1%), 31-40: 320 (30.2%), 41-50: 199 (18.8%), 51-60: 63 (6.0%), 61-70: 18 (1.7%), 71+: 2 (0.2%) | 70.9 | Intervention arm 1: iPST - an app based on problem solving therapy, Intervention arm 2: Project Evo - a therapeutic video game meant to improve cognitive skills associated with depression, Intervention arm 3: Health Tips - an app that provides information about strategies to improve mood. | As per intervention column | Mixed sample – participants recruited via multiple settings. | Active ambulatory assessment: PHQ-9, SDS , Global Impression of Change Scale, 3 question sleep assessment, Mental Health Services Used - weekly. PHQ-2 - daily. Passive ambulatory assessment - time of call/SMS, call duration, SMS length, activity type, distance travelled. | 12w | PHQ-9, SDS, GAD-7, AUDIT-C, IMPACT Mania & Psychosis — baseline |  |
| Dai et al 2022 | USA | 106 | Intervention: 46.86 (12.1), Control: 47.47 (12.88) | Intervention: 76.2%, Control: 76.6% | I-CARE2: Integrated Coaching for Better Mood and Weight. Includes PEARLS program for collaborative stepped depression care, which uses PST augmented with behavioural activation. | TAU. In addition they receive a summary of behavioural health and weight management services at UI health and a Fitbit. | Secondary care diabetes patients who were also depressed | Wearable activity tracker (Fitbit) measuring sedentary minutes, lightly active minutes, minutes of heart rate zone in fat-burn, minutes of heart rate zone in cardio, total walking distance, activity calories, minutes awake in main sleep, restless count in main sleep, efficiency in main sleep, time in bed of main sleep. | 6 months | PHQ-9, CAGE-AID - baseline. Emotional Regulation Questionnaire, COPE Inventory for cognitive coping, BRISC questionnaire of emotional resilience and self-efficacy, Clutter Image Rating Scale, SDS, PCL-C, PROMIS SF, PSS, MINI, GAD-7, SCL-20 - baseline, 2 months, 6 months (57) |  |
| Tonning et al 2021 | Denmark | 120 | Intervention: 44.5 (14.0), Control: 43.4 (14.3) | Intervention: 47.5 (28), Control: 43.4 (14.3) | Monsenso system plus: 1. study nurse reviewing data and contacting patients if sign of deterioration to offer advice 2. self-monitored data graphically visualised 3. smartphone based CBT modules | TAU | Tertiary care – specialist mood disorder service for patients with a new diagnosis of bipolar or treatment resistance | Daily smartphone self monitoring items - mood, sleep duration, medication taken, activity, irritability, mixed mood, cognitive problems, alcohol consumption, stress, menstruation, individualised EWS, anxiety, self-defined personal parameters, free-text note.                     Objective smartphone data - phone usage, social activity, step count, GPS location | 6 months | Rate and accumulated duration of psychiatric readmissions. HDRS-17, FAST, PSS, WHOQoL-BREF, BDI, HDRS-6, RAS, MARS, Roger’s Empowerment Scale, WHO5, RRS, PSWQ, BADS, VSS-A - baseline, months 3 and 6 |  |
| Frank et al 2022 | USA | 133 | 32.94 (11.39) | 73.68 | Passive behaviour monitoring, 10 psychoeducation modules, Cue system providing personalised micro-interventions delivered every 2/3 days, self-report completion | Passive behaviour monitoring, self-report completion | Secondary care - academic psychiatry centre | Active ambulatory assessment: daily VAS rating of mood. Passive ambulatory assessment: step-count, activity classification, GPS data, screen use data and battery time. | 16w | PHQ-8 - fortnightly |  |
| Hunkeler et al 2012 | USA | 103 | Intervention: 48.49 (12.83), Usual care: 51.88 (10.56) | 79.6 | eCare for Moods - website offering personalised self-monitoring, messaging with eCare manager, depression psychoeducation, CBT modules, online discussion group, problem-specific advice, personal database, task lists, appointment calendar. | TAU | Secondary care | Personalised self-monitoring via eCare for Moods - tracking health-related disability, medication adherence, side effects, alcohol and drug use, new symptoms, early warning signs. Graphs of monitoring data displayed over time. | 12 months | SCID - baseline, SDS, SF36, AUDIT, custom social support questionnaire, custom care satisfaction questionnaire - 3 monthly |  |
| Klein et al 2016 | Germany | 1013 | Intervention: 42.8 (11.0), Control: 42.9 (11.0) | 69 | Deprexis self-help program | TAU | Mixed sample – participants recruited via multiple settings. | Weekly contact by trained email supporter - short feedback based on participants’ program usage over the past week. Feedback is relatively generic in nature and does not apply therapeutic strategies in detail. PHQ-9 biweekly. | 12w | PHQ-9, HDRS-24, QIDS-C16, MINI, Structured assessment of suicidality, SF-12, FEP-2 - baseline, 3 months, 6 months |  |
| Tuvey et al 2023 | USA | 53 | SM: 40.11 (10.65), SM + Coach: 47.5 (14.26), AC: 49.55 (14.74) | SM: 76, SM + Coach: 77, AC: 61 | Intervention arm 1: Annie Secure Messaging (SM) system, Intervention arm 2: Annie SM system with coaching | Attention control – psychoeducation materials plus psychoeducation discussion at baseline with staff member | Primary care - veterans | Intervention arm 1: Annie Secure Messaging (SM) system – promoting antidepressant adherence with texts around efficiacy and AEs, Intervention arm 2: Annie SM system with coaching – weekly coaching call with a study member to review weekly data reported via Annie looking at trends over time – participants asked to reflect on trends and identify any self-management issues | 12w | MARS, PHQ-9, FIBSER – baseline, 6, 12 weeks |  |
| White et al 2023 | UK | 100 | 53.3 (14.3) | 76 | RADAR-base app plus addition in-app components grounded in behavioural theory and using the Capability, Opportunity and Motivation framework of behaviour change. The app used symptom tracking, behavioural feedback via progess visualisation and instant access to researcher contact details. | RADAR-base app | Mixed sample – participants recruited via multiple settings e.g clinician referral, advertising. | PHQ-8, RSES, speech task – weekly. Heartrate, step-count, GPS location, app usage metrics – continuous via Fitbit/RADAR-base app | 12w | LIDAS - baseline, IDS-SR, CIDI-SF, GAD-7, WSAS, BIPQ, Life Events, CSRI, UES, ESQ, MAUQ – baseline and 12 weeks. |  |
| **Included randomised controlled trials in people with bipolar disorder** | | | | | | | | | | | |
| **Study** | **Country** | **Sample** | **n** | **Mean age in years (SD)** | **% female** | **Intervention** | **Comparator** | **Setting** | **Ambulatory assessment /mood tracking protocol** | **Ambulatory assessment duration** | **Established mood outcome** |
| Bilderbeck et al 2016 | UK | Bipolar 1: 65.3%, Bipolar 2: 34.7% | 121 | 44 (1) | 72.7 | Facilitated Integrated Mood Management (therapist administered psychoeducation) | Manualised Integrated Mood Management (self-administered psychoeducation) | Primary care – not in current mood episode | QIDS-SR-16, ASRM - administered weekly via TrueColours | 12 months | QIDS-SR-16, ASRM – weekly |
| Denicoff et al 2002 | USA | Bipolar 1: 63%, Bipolar 2: 37% | 52 | 41.3 (11.4) | 52 | year 1: lithium, year 2: switch to carbamazepine, year 3: lithium & carbamazepine | year 1: carbamazepine, year 2: switch to lithium, year 3: lithium & carbamazepine | Tertiary care – specialist mood disorder service with the majority of patients experiencing rapid cycling | NIMH Life Chart Methodology - prospective —twice daily mood self-rating | 3 years | HAM-D, IDS, YMRS, CGI-BP – monthly |
| Faurholt-Jepsen et al 2015 | Denmark | Bipolar 1: 67%, Bipolar 2: 33% | 67 | 29.3 (8.43) | 67 | MONARCA system plus: 1. study nurse reviewing data and contacting patients if sign of deterioration to offer advice 2. self-monitored data graphically visualised | Normal smartphone use | Tertiary care – specialist mood disorder service for patients with a new diagnosis of bipolar or treatment resistance | Daily smartphone self monitoring - mood, sleep duration, medication taken, activity, irritability, mixed mood, cognitive problems, alcohol consumption, stress, menstruation, individualised EWS | 6 months | HAMD, YMRS, PSS, FAST - monthly |
| Faurholt-Jepsen et al 2019 | Denmark | Bipolar 1: 59%, Bipolar 2: 41% | 129 | 43 (12) | 59 | Monsenso system plus: 1. study nurse reviewing data and contacting patients if sign of deterioration to offer advice 2. self-monitored data graphically visualised | Normal smartphone use | Tertiary care – specialist mood disorder service for patients with a new diagnosis of bipolar or treatment resistance | Daily smartphone self monitoring items - mood, sleep duration, medication taken, activity, irritability, mixed mood, cognitive problems, alcohol consumption, stress, menstruation, individualised EWS, anxiety, self-defined personal parameters, free-text note.                     Objective smartphone data - phone usage, social activity, step count, GPS location | 9 months | HDRS-17, YMRS, FAST, PSS, WHOQoL-BREF, BDI, ASRM, MARS Roger’s Empowerment Scale - baseline, months 1, 3, 6, 9 |
| Faurholt-Jepsen et al 2020 | Denmark | Bipolar 1: 58%, Bipolar 2: 42% | 98 | 42.69 (13.46) | 52 | Monsenso system plus: 1. study nurse reviewing data and contacting patients if sign of deterioration to offer advice 2. self-monitored data graphically visualised | Usual care | Tertiary care – specialist mood disorder service for patients with a new diagnosis of bipolar or treatment resistance | Daily smartphone self monitoring items - mood, sleep duration, medication taken, activity, irritability, mixed mood, cognitive problems, alcohol consumption, stress, menstruation, individualised EWS, anxiety, self-defined personal parameters, free-text note.                     Objective smartphone data - phone usage, social activity, step count, GPS location | 6 months | HDRS-17, YMRS, FAST, PSS, WHOQoL-BREF, BDI, HDRS-6, ASRM, RAS, MARS Roger’s Empowerment Scale, RRS, PSWQ, BAS, VSS-A - baseline, months 3 and 6 |
| Gliddon et al. 2018 | Austrailia & USA | Bipolar 1: 55%, Bipolar 2: 38% | 304 | 39.47 (11.19) | 82 | Intervention 1: Discussion forum plus MoodSwings-Plus: MoodSwngs plus additional CBT-based interactive elements – tools to support mood and medication monitoring, life-chart development, cognitive strategies, motivational interviewing techniques, self reflection, problem solving, identification of personal triggers and a relapse prevention plan.    Intervention 2: Discussion forum plus MoodSwings: Online intervention comprising: mood monitoring, assessing prodromal mood states, preventing relapse, setting SMART goals. Online delivery of MAPS (Mood Assessment Prevent SMART) programme. | Discussion forum | Mixed sample – participants recruited via advertising. | Online mood-monitoring via MoodSwings & MoodSwings-Plus websites | 12 months | MADRS, YMRS, SF-12, Q-LES-Q, MARS, TIME – baseline, 3, 6, 9, 12 months. |
| Lauder et al 2015 | Australia | Bipolar 1: 52%, Bipolar 2: 48% | 156 | MoodSwings-Plus: 39.87 (11.26), MoodSwings: 41.35 (9.85) | 62 | MoodSwings-Plus: MoodSwings plus additional CBT-based interactive elements – tools to support mood and medication monitoring, life-chart development, cognitive strategies, motivational interviewing techniques, self reflection, problem solving, identification of personal triggers and a relapse prevention plan. | MoodSwings: Online intervention comprising: mood monitoring, assessing prodromal mood states, preventing relapse, setting SMART goals. Online delivery of MAPS (Mood Assessment Prevent SMART) programme. | Mixed sample – participants recruited via clinician referral, advertising. | Online mood-monitoring via MoodSwings & MoodSwings-Plus websites | 12 months | ASRM, MADRS-S, MOS-SSS, Levenson's Internal, Powerful Others and Chance Locus of Control scale, MARS, Exploratory Global Assessment Measures, GSEVDEP, GSEVMANIA, GPF:Depression, GPF: Mania, GQOL, SCID, Self-report Relapse – Baseline, 3, 6, 12 months. |
| Castle et al 2018 | Australia | Bipolar 1: 71%, Bipolar 2: 28% | 84 | Control group: 42.6 (11.3), Treatment group: 41.6 (11.0) | 84 | Structured group programme comprising an initial block of 12 weekly sessions with 3 additional monthly booster sessions to support participants in applying knowledge and skills to their lives. Included weekly telephone calls to remind participants of the next group session and to offer support for homework tasks. | Usual care plus weekly telephone calls | Mixed sample – participants recruited via clinician referral, advertising. | Weekly telephone calls – weekly for 12 weeks. | 12 months | Structured telephone interview to determine relapse – monthly for 12 months, MADRS, YMRS – baseline, 3, 12 months. |
| Goulding et al 2022 | USA | Bipolar 1: 100%, Bipolar 2: 0% | 205 | 42 (12) | 61 | Livewell | Usual care | Secondary care – 1 previous mood episode in the past year and current care by psychiatrist/nurse practitioner. | Smartphone based self management intervention - daily and weekly check-ins for weeks 1-16. Daily - adherence, sleep, duration, routine, wellness levels. Weekly - symptom severity scoring for all individual DSM-IV mood symptoms. | 4 months | QIDS, YMRS, WHOQOL-BREF - baseline, months 2, 4, 8, 10, 11 |
| Petzold et al. 2019 | Germany | Bipolar 1: 71%, Bipolar 2: 39% | 73 | Intervention: 44.32 (11.63), Control: 42.69 (12.34) | 45 | 6 weekly group Psychoeducation sessions plus 54 weeks of ChronoRecord | 6 weekly supportive non-structured group meetings plus 54 weeks of daily unstructured computer-based self-reports/diary | Secondary care – remission for 2 months but 1 mood episode in the past 3 years. | ChronoRecord - daily mood, sleep, life events, menstrual data, psychiatric medication, weekly – weight. | 12.5 months | SCID, YMRS, HDRS-17 - baseline, 1.5 months, then every 3 months up to 24 months. SF-36, GSE, HLOC, PICS - baseline, 1.5, 6, 12, 15, 24 months |
| Van den Berg et al 2023 | Netherlands | Bipolar 1: 50%, Bipolar 2: 50% | 62 | Intervention: 46.5 (11.1), Control: 42.73 (13.0) | 58 | Imagery Focussed CBT | Group Psychoeducation | Mixed sample – participants recruited via advertising. | NIMH Life Chart Methodology - daily mood and anxiety self-rating. | 4 months | QIDS-SR, ASRM, BAI, ALS-18, Life-Rift, BHS, VAS Imagery, MICQ-BD – weekly |
| Goldberg et al. 2006 | USA | Bipolar 1: 70%, Bipolar 2: 30% | 177 | Intervention: 38.5 (9.1), Control 37.7 (10.5) | 56.5 | Lamotrogine monotherapy | Placebo | Tertiary care – specialist mood disorder service with past year rapid cycling. | NIMH Life Chart Methodology - daily mood self-rating | 6.5 months | NIMH Life Chart Methodology - nil other |
| Langosch et al 2008 | Germany | Bipolar 1: 50%, Bipolar 2: 50% | 44 | Quetiapine: 45.4 (11), Valproate 37.8 (13.8) | 60 | Quetiapine monotherapy | Valproate monotherapy | Tertiary care – specialist mood disorder service with past year rapid cycling. | NIMH Life Chart Methodology - daily mood self-rating | 12 months | CGI-BP-II, HDRS, MADRS, YMRS, SAS - weekly for 1st 6 weeks, then monthly until 12 months |
| Leverich et al 2006 | USA | Bipolar 1: 72.3, Bipolar 2: 26.4 | 159 | 41.6 (12.2) | 47.8 | Buproprion or sertraline or venlafaxine as an adjunct to mood stabilisers | Buproprion or sertraline or venlafaxine as an adjunct to mood stabilisers | Tertiary care – specialist mood disorder service | NIMH Life Chart Methodology - daily mood self-rating | 12 months | CGI-BP – unclear frequency |
| Lieberman et al 2010 | USA | Bipolar 1: 13%, Bipolar 2: 71%, Bipolar NOS: 16% | 48 | Paper chart: 39.5 (12.9), Online chart: 35.8 (12.0) | 75 | NIMH Life Chart Methodology - prospective — daily mood self-rating | Online Life Chart adaptation | Secodary care -  currently under the care of psychiatrist/nurse practitioner | NIMH Life Chart Methodology - prospective — daily mood self-rating / online Life Chart adaptation | 3 months | NIMH Life Chart Methodology - nil else |
| Depp et al 2012 | USA | Bipolar 1: 90%, Bipolar 2: 10% | 56 | Paper chart: 46.1 (13.5), Phone chart: 44.0 (14.0) | 57.5 | Paper and pen life charting | Smartphone based life charting | Mixed sample – participants recruited via advertising. | NIMH Life Chart Methodology - prospective — daily mood self-rating via paper and pen and via smartphone | 3 months | MADRS, YMRS - baseline, 6 weeks, 12 weeks. RBANS - baseline. |
| Pahwa et al 2023 | USA | Bipolar 1: 100% | 122 | 43.75 (14.05) | 68.60 | KIOS app | eMoods app | Tertiary care | KIOS app - self report assessment of 8 different symptoms e.g sadness/pessimism and delivery of guidance in relation to symptom change.    eMoods app – self report mood and symptoms diary tracking daily outlook, motivation, habits, sleep, medications etc | 52 weeks | BISS - monthly |
| **Included non-randomised studies in people with bipolar disorder** | | | | | | | | | | |  |
| Study | Country | Sample | n | Mean age in years (SD) | % female | Intervention | Setting | Ambulatory assessment /mood tracking procedure | Ambulatory assessment duration | Established mood Outcome |  |
| Anzy et al 2021 | Czech Republic | Not reporrted | 99 | 37.1 (11) | 61 | Aktibipo self-rating questionnaire | Not reported | ASERT mood self-reports weekly - 10 items that map depressive, manic and nonspecific symptoms on a likert scale. | 18 months | MADRS, YMRS |  |
| Hidalgo-Mazzei et al 2016 | Spain | Bipolar 1: 67.3, Bipolar 2: 26.5 | 51 | 43.92 (11.36) | 42.9 | SIMPLe 1.0 - Self-report 5 item test assessing mood, energy, sleep duration, medication adherence and irritability - daily. DSM-5 criteria for manic/depressive episodes - weekly. Daily notification of psychoeducation relapse prevention message (500 different messages possible) targeting specific situations based on ambulatory assessment data. | Tertiary care - mood disorders service | Self-report 5 item test assessing mood, energy, sleep duration, medication adherence and irritability - daily. DSM-5 criteria for manic/depressive episodes - weekly. | 3 months | HDRS, YMRS, FAST, Morisky-Green 8-item test - baseline, 3 months |  |
| Hidalgo-Mazzei et al 2018 | Spain, Argentina | Not reported | 201 | 36.59 (11) | 63.2 | SIMPLe 1.5 - Self-report 5 item test assessing mood, energy, sleep duration, medication adherence and irritability - daily. DSM-5 criteria for manic/depressive episodes - weekly. Daily notification of psychoeducation relapse prevention message (500 different messages possible) targeting specific situations based on ambulatory assessment data. Additional app components/modules: medication reminders, personalised prodromal symptoms, gamification module, mood-chart sharing, psychoeducational messages community. | Tertiary care - mood disorders service | Self-report 5 item test assessing mood, energy, sleep duration, medication adherence and irritability - daily. DSM-5 criteria for manic/depressive episodes - weekly. | 12 months | MDQ - baseline, WHO-5, SF-36 - baseline, 6 months |  |
| Garcia-Estela et al 2022 | Spain, Chile, Argentina, Mexico, Colombia, Guatambulatory assessmentla, Brazil, Other | Not reported | 503 | 34.74 (10.48) | 67.7 | SIMPLe 1.5 - Self-report 5 item test assessing mood, energy, sleep duration, medication adherence and irritability - daily. DSM-5 criteria for manic/depressive episodes - weekly. Daily notification of psychoeducation relapse prevention message (500 different messages possible) targeting specific situations. Additional app components/modules: medication reminders, personalised prodromal symptoms, gamification module, mood-chart sharing, psychoeducational messages community. | Mixed sample – participants recruited online. | Self-report 5 item test assessing mood, energy, sleep duration, medication adherence and irritability - daily. DSM-5 criteria for manic/depressive episodes - weekly. | 6 months | MDQ - baseline. WHO-5 - baseline, 6 months. Satisfaction and perceived helpfulness, System Usability Scale - 6 months. |  |
| Bauer et al 2023 | Germany, USA | Bipolar 1: 55.0%, Bipolar 2: 31.5%, Bipolar NOS: 5.1, Unipolar: 8.4 | 609 | 40.3 (11.8) | 71.4 | ChronoRecord - daily mood via 100 point VAS, sleep, life events, menstrual data, psychiatric medication, weekly - weight. | Secondary care - outpatient | ChronoRecord - daily mood via 100 point VAS, sleep, life events, menstrual data, psychiatric medication, weekly - weight. | Average follow up: 227 days | Varied between studies and not reported in summary paper |  |
| Bos et al 2022 | The Netherlands | Bipolar 1: 40%, Bipolar 2: 60% | 20 | 20-35 years: n = 9, 36-50 years: n = 8, 51-65 years: n = 3 | 80 | 5x EMA smartphone assessments daily - 29 items assessing monetary mood, symptoms, sleep and activities. Weekly ASRM, QIDS-SR-16 delivered via RoQua platform. | Tertiary care - mood disorders service | 5x EMA smartphone assessments daily - 29 items assessing monetary mood, symptoms, sleep and activities. Weekly ASRM, QIDS-SR-16 delivered via RoQua platform. | 4 months (range: 16-32 weeks) | ASRM, QIDS-SR-16 - weekly |  |
| Bowden et al 2021 | USA | Not reported | 20 | Not reported | Not reported | KIOS app - self report assessment of 8 different symptoms e.g sadness/pessimism and delivery of guidance in relation to symptom change. | Tertiary care - mood disorders service | KIOS app - self report assessment of 8 different symptoms e.g sadness/pessimism and delivery of guidance in relation to symptom change. | 3 months | SCID - baseline. Nil other validated measures. |  |
| Dominiak et al 2022 | Poland | Bipolar 1: 61%, Bipolar 2: 39% | 84 | 36.2 (9.5) | 55 | BDmon app - Passive ambulatory assessment: phone/SMS logs, participant speech information extracted from daily phone calls, Active ambulatory assessment: self-report mood | Mixed sample – participants recruited via inpatient/outpatient services | BDmon app - Passive ambulatory assessment: phone/SMS logs, participant speech information extracted from daily phone calls, Active ambulatory assessment: self-report mood | mean: 208 days (SD: 32) | HDRS-17, YMRS - 2 weekly |  |
| Emden et al 2021 | Germany | Not reported | Total: 997, Depression: 409, Bipolar Disorder: 48, Anxiety: 58, Psychosis: 21, Healthy controls: 458 | 35.99 (13.57) | 67.3 | ReMAP system | Unclear - ReMAP offered to a variety of participants already enrolled in multiple longitudinal studies | ReMAP system - Active ambulatory assessment - single mood likert scale, single item sleep scale assessing self-report sleep time, voice sample - weekly. Passive ambulatory assessment: step-count, GPS location, accelerometer - continuous. | 12 months | BDI - 2 weekly |  |
| Stanislaus et al 2020 | Denmark | Bipolar 1: 33.5, Bipolar 2: 66.5 | Bipolar Disorder: 203, Unaffected first-degree relatives: 54, HC: 109 | Median: 28 (IQR: 24-35) | 69 | Monsenso system | Tertiary care - mood disorders service | Monsenso system - Active ambulatory assessment: daily smartphone self monitoring items - mood and activity level, HDRS-17 & YMRS every 3 days. Passive ambulatory assessment: Objective smartphone data - phone usage, call/SMS logs, step count | Median 106 days (IQR: 48-204) | SCAN, HDRS-17, YMRS, FAST, IPAQ - baseline and yearly |  |
| Lee et al 2022 | South Korea | Bipolar 1: 28.9, Bipolar 2: 35.9, Depression: 35.2 | Total: 495, Depression: 95, Bipolar Disorder: 175 | Subjects included in analysis: 23.3 (3.63), subjects not included in analysis: 22.8 (3.10) | Subjects included in analysis: 54.4, subjects not included in analysis: 59.1 | eMoodChart system | Secondary care | eMoodChart system - Active ambulatory assessment: self report daily mood and energy, . Passive ambulatory assessment: Fitbit measuring step-count, heart rate, sleep, ambient light (android online) | mean: 279.7 days (SD: 263.5), median: 505 days (range: 72-1515) | Nil other measures |  |
| Born et al 2014 | Germany | Bipolar 1: 72.2, Bipolar 2: 25.9 | 108 | 39.3 (13.3) | 47.2 | NIMH Life Chart Methodology - prospective | Tertiary care - mood disorders service | NIMH Life Chart Methodology - prospective —twice daily mood self-rating | Unclear - potentially 3 years | YMRS, IDS-C, CGI-BP - monthly |  |
| Lieberman et al 2011 | USA | Not reported | 64 | Not reported | Not reported | MoodChart | Mixed sample – participants recruited online. | MoodChart - daily self report mood via email/online, Social Rhythm Metric - activity level over the previous 7 days | mean: 84 (range: 42-90) | Nil other measures |  |
| Kupka et al 2005 | USA, Netherlands, Germany | Bipolar 1: 77.7, Bipolar 2: 19.3 | 539 | 42.1 (11.5) | 56 | NIMH Life Chart Methodology - prospective | Tertiary care - mood disorders service | NIMH Life Chart Methodology - prospective — daily mood self-rating | 1 year | Nil other measures |  |
| O’Rourke et al 2021 | Canada, USA, UK, South Africa, Australia | Not reported | 50 | 50% >45 years | Not reported | Twice daily Bipolar Disorder Symptom Scale | Mixed sample – participants recruited online. | Twice daily Bipolar Disorder Symptom Scale | 4+ months | Nil other measures |  |
| Tseng et al 2022 | Taiwan | Not reported | 159 | 34.5 (11.34) | 55.97 | Smartphone app collecting daily/weekly active and passive ambulatory assessment. | Secondary care - outpatients | Active ambulatory assessment: daily mood, sleep duration. Weekly ASRM, DASS-21. Passive ambulatory assessment: GPS location | number of days on which participants performed self-assessments - mean: 94.25 days (median 52.5, range 2 to 398) | HDRS, YMRS, DASS-21, PSQI - baseline |  |
| Ebner-Priemer et al 2020 | Germany | Bipolar 1: 58.6, Bipolar 2: 41.4 | 31 | 44 (11.9) | 55 | MovisensXS | Tertiary care - mood disorders service | MovisensXS system - Active ambulatory assessment: self-report mood, sleep diary.  Passive ambulatory assessment: call/text logs, GPS data, velocity, step-count. | 12 months | SCID-I section A, YMRS, BRMRS, MADRS - 2 weekly. |  |
| Gideon et al 2016 | USA | All participants had rapid cycling | 37 | Not reported | Not reported | PRIORI app | Secondary care - outpatients and inpatients | PRIORI system - records speech made on telephone calls. Weekly HDRS, YMRS. | 6-12 months. Mean: 29.2 weeks (SD: 16.4) | HDRS, YMRS - weekly |  |
| Schneider et al 2022 | Czech Republic | Bipolar 1: 64, Bipolar 2: 36 | Bipolar disorder: 35, HC: 26 | 39.75 (SD: 12.85) | 60 | MINDPAX - actigraphy | Tertiary care - mood disorders service | MINDPAX - actigraphy measuring sleep data | 3 months | MINI, MADRS, YMRS - baseline |  |
| Scharer et al 2015 | Germany | Not reported | 54 | 40.6 | 46.3 | PLC app | Mixed sample – participants recruited via advertising | PLC app - daily self report mood | 18 months | IDS-C, YMRS - monthly (mean: 32 days, median: 23 days, range: 2 to 205 days) |  |
| van den Heuvel et al 2018 | Netherlands | Bipolar 1: 76.7, Bipolar 2: 23.3 | 66 | 45.17 (10.67) | 66.7 | PHR-BD system | Tertiary care - mood disorders service | PHR-BD system - including 9 modules covering: medical record, medication, treatment and medical passport, general information about BD, medical results/reports, platform to send messages to appointed clinician, mood chart with daily self report mood, personal crisis plan. | 12 months | QBL-NL, OQ-45.2, ASRM, IDS-SR, MANSA, NEL - baseline, 12 months |  |
| Arribas et al 2018 | UK | Bipolar Disorder: 53, Borderline Personality Disorder: 33, HC: 53 | 139 | Bipolar Disorder: 38 (+/-21), Borderline Personality Disorder: 34 (+/-15), HC:37 (+/-20) | Bipolar Disorder: 69.8%, Borderline Personality Disorder: 94%, HC: 66% | AMoSS study system | Mixed sample – participants recruited via advertising | AMoSS study system - Active ambulatory assessment: daily mood rating across categories of anxiety, elation, sadness, anger, irritability and energy using MoodZoom questionnaire, ASRM, QIDS-SR16, EQ-5D, GAD-7 assessed weekly. Passive ambulatory assessment: GPS, actigraphy, ambient light, call/SMS logs, heart rate via smartphone/Fitbit/GENEActive accelerometer/Proteus patch (heart rate data only for one week). | 3 months, with 61 participants continuing for 12 months | KEAS, IPDE - baseline, ASRM, QIDS-SR16, EQ-5D, GAD-7 - weekly |  |
| Lewis et al 2023 | UK | Bipolar 1: 61.6, Bipolar 2: 38.4 | 649 | 53 (range: 22-83) | 68 | Bipolar Disorder Research Network using True Colours | Mixed sample – participants recruited via secondary care/advertising | Bipolar Disorder Research Network using True Colours: weekly ASRM/QIDS-16SR | 21 months | SCAN - baseline, ASRM/QIDS-SR16 weekly |  |
| McKnight et al 2017 | UK | Bipolar 1: 62.6, Bipolar 2: 33.3 | 367 | 41 (SD: 13.7, range: 16-76) | 66.7 | OXTET-1 using True Colours | Mixed sample – participants recruited via secondary care/advertising | OXTET-1 using True Colours - ASRM/QIDS-SR16 delivered via weekly SMS/email | 27.5 ± 22.5 months (range: 1-81) | Clinical interview to confirm diagnosis - baseline, ASRM/QIDS-SR16 weekly |  |
| Ortiz et al 2023 | Canada | Bipolar 1: 60.9, Bipolar 2: 39.1 | 87 | 38.9 (SD: 12.4) | 67.8 | E-monitoring system | Secondary care | E-monitoring system - Active ambulatory assessment: daily rating of mood, anxiety, energy level using e-VAS. Weekly: PHQ-9, ASRM. Passive ambulatory assessment: Oura Health Oy 3d accelerometer/hyroscope measuring activity, sleep, Infrared optical pulse measuring heart rate, heart rate variability | 229.4 days (± 12.4) | Weekly PHQ-9, ASRM |  |
| **Included non-randomised studies in people with depression** | | | | | | | | | |  |  |
| **Study** | **Country** | **n** | **Mean age in years (SD)** | **% female** | **Intervention** | **Setting** | **Ambulatory assessment/mood tracking protocol** | **Ambulatory assessment duration** | **Established mood outcome** |  |  |
| Aguilera et al 2017 (17) | USA | 91 | Text message group: 51.71 (11.55), Control group: 51.83 (11.73) | 78.8 | Group CBT with text messaging intervention (HealthySMS), Group CBT without text messaging intervention | Secondary care | HealthySMS: up to 5 automated text messages including: daily mood rating, supplementation of therapy content, weekly reminders to attend CBT sessions, monthly opt out messages to end message delivery if desired. Weekly PHQ-9 for 16 weeks. | median: 13.5 weeks | PHQ-9 |  |  |
| Aikens et al 201 5(18) | USA | 221 | 51.4 (12.7) | 78.6 | Automated Interactive Voice Response telephone calls | Primary care | Automated Interactive Voice Response telephone calls assessing weekly symptom severity - PHQ-9 and antidepressant adherence. | 6 months | PHQ-9 |  |  |
| Benedyk et al 2023 (19) | Germany | Total: 65, Depression: 24, Schizophrenia: 20, Healthy Controls: 21 | Schizophrenia group: 34.6 (10.3), Depression group: 38.0 (13.5), Healthy controls: 38.5 (11.1) | Schizophrenia group: 75%, Depression group: 62.5%, Healthy controls: 48% | INDICATE-N/Movisens GmbH system | Secondary care | INDICATE-N/Movisens GmbH system. Active ambulatory assessment: e-diary: twice daily fixed time smartphone-based self-ratings of mood, affect and social context via 21 e-diary items. Mood assessed via six item Multidimensional Mood Questionnaire. Participants also completed 3 intense EMA phases with 6 daily prompts for 10 days each.                                   Passive ambulatory assessment: Step count, real-life GPS location tracking. | 6 months | Mini-DIPS Neuropsychiatric Interview, PANSS, MADRS, BDI, SNS, CGI - baseline, 3, 6 months |  |  |
| Bonilla-Escribano et al 2023 (20) | Spain/France | Total: 275, PTSD: 74, Depression: 40, Binge Eating Disorder: 23, Agoraphobia: 18, AUD: 9, Bipolar Disorder: 6, OCD: 7, Panic Disorder: 26, Social Anxiety Disorder: 4, SUD (non-alcohol): 5 | 40 (14) | 67.27 | Memind & EB2 apps | Secondary care | Memind app: 4 question daily ambulatory assessment inspired by the Salzburg Suicide Process Questionnaire. 4 questions pulled from a 32 item pool. | 6 months | IDS, C-SSRS, MARS, CAGE, FTND, LTE, ISI, CTQ, BIS |  |  |
| Carpenter et al 2021 (21) | USA | 20 | 47.1 | 80 | Maintenance TMS | Secondary care | Weekly PHQ-9 delivered via app | 12 months | PHQ-9 |  |  |
| de Angel et al 2023 (22) | UK | 66 | 34.6 (11.1) | 61 | Psychotherapy | Primary care - IAPT services | RADAR-base system PHQ-8, RSES, speech task – weekly. Heartrate, step-count, GPS location, acceleration, light levels, phone interaction, nearby Bluetooth device detection, battery level, weather, sleep, app usage metrics – continuous via Fitbit/RADAR-base app. | 7 months | THINC-it app - cognitive tasks monthly: One-back Test, Trail Making Test Part B, Digit Symbol Substitution Test, Choice Reaction Time Task, Perceived Deficits Questionnaire. PHQ-9, GAD-7, QIDS-SR16, WSAS via REDCap - 3 monthly. |  |  |
| Drake et al 2013 (42) | UK | 20 | Completers: 38.9 (12.6), Dropouts: 32.8 (15.3) | 75 | Moodscope website | Primary care | PHQ-9, GAD-7 - weekly. Moodscape self-rated mood - selecting which of each 20 interactive mood-adjective playing cards describes current mood. | 3 months | PHQ-9, GAD-7 - weekly |  |  |
| Emden et al 2021 (24) | Germany | Total: 997, Depression: 409, BD: 48, Anxiety: 58, Psychosis: 21, Healthy controls: 458 | 35.99 (13.57) | 67.3 | ReMAP system | Unclear - ReMAP offered to a variety of participants already enrolled in multiple longitudinal studies | ReMAP system - Active ambulatory assessment - single mood likert scale, single item sleep scale assessing self-report sleep time, voice sample - weekly. Passive ambulatory assessment: step-count, GPS location, accelerometer - continuous. | 12 months | BDI - 2 weekly |  |  |
| Funkhouser et al 2024 (25) | USA | 90 | 16.57 (1.43) | 63 | EARS app | Secondary care | EARS app - Active: daily mood rating. Passive: keyboard inputs. | 12 months | K-SADS, PSR |  |  |
| Helmich et al 2022 (26) | Netherlands | 41 | 40.1 (14.4) | 85 | TRANS-ID Recovery System | Primary/secondary care | TRANS-ID Recovery System - 27 item EMA delivered 5 times a day about current feelings, activities and surroundings incorporating VAS. Weekly 14 item depressive symptom scale of SCL-90. Weekly rating of how much depressive symptoms have bothered them. | 4-6 months | IDS-SR, Weekly 14 item depressive symptom scale of SCL-90 |  |  |
| Janevic et al 2016 (27) | USA | 32 | 58 (13) | 78 | IVR | Primary care | Automated Interactive Voice Response telephone calls assessing symptom severity - 6 item version of the 17 item HAM-D, PGI, WSA - and offering tailored treatment recommendations and individualised feedback. | 14 weeks | PHQ-8, PHQ-9, SDS |  |  |
| Kathan et al 2022 (43) | Germany | 48 | Not reported | Not reported | MAIKI system | Not reported | MAIKI system - Passive ambulatory assessment: app sessions and app useage, metadata on general phone settings and phone actions, GPS data, communication information e.g contacts, call and SMS data. Active ambulatory assessment - GAD-7, PSS, PHQ-9 - weekly, items extracted from the CESD, PDD, PSQI, PHQ-2 - daily | 3 months | Audio data from interview with clinical psychologist, AQoL-8D, BADSSF, Big Five Inventory - baseline. |  |  |
| Kline et al 2024 (29) | USA | 207 | 38.74 (10.42) | 81.6 | MLife app | Mixed sample – participants recruited via multiple settings e.g advertising. | MLife app. Active EMA: 3 times daily modified PHQ-9 assessing symptoms over previous 4 hours, VAS of mood, optional diary entry. | 3 months | SCID |  |  |
| Lee et al 2022 (44) | South Korea | Total: 495, MDD: 95, BD: 175 | Subjects included in analysis: 23.3 (3.63), subjects not included in analysis: 22.8 (3.10) | Subjects included in analysis: 54.4, subjects not included in analysis: 59.1 | eMoodChart system | Secondary care | eMoodChart system - Active ambulatory assessment: self report daily mood and energy, . Passive ambulatory assessment: Fitbit measuring step-count, heart rate, sleep, ambient light (android online) | mean: 279.7 days (SD: 263.5), median: 505 days (range: 72-1515) | Nil other measures |  |  |
| Matcham et al 2022 (30) | UK, Spain, Netherlands | 623 | 46.4 (15.3) | 75.6 | RADAR-base system | Mixed sample – participants recruited via multiple settings e.g primary care, secondary care, advertising. | RADAR-base system PHQ-8, RSES, speech task – weekly. Heartrate, step-count, GPS location, acceleration, light levels, phone interaction, nearby Bluetooth device detection, battery level, weather, sleep, app usage metrics – continuous via Fitbit/RADAR-base app. | 2 years | THINC-it app - cognitive tasks 6 weekly: One-back Test, Trail Making Test Part B, Digit Symbol Substitution Test, Choice Reaction Time Task, Perceived Deficits Questionnaire. PHQ-9, GAD-7, QIDS-SR16, WSAS via REDCap - 3 monthly. |  |  |
| Mcintyre et al 2021 (31) | Canada | 523 | 46 (12.7) | ~76 exact numbers not reported | Mind.me system | Mixed sample – participants recruited via multiple settings e.g advertising. | Mind.me system - Passive ambulatory assessment: daily call/SMS count, location variance, normalised entropy, number of GPS geolocation coordinate clusters, total distance (km), mean absolute deviation in distance (km). | 3 months | PHQ-9 - monthly |  |  |
| Stamatis et al 2024 (64) | USA | 1013 | 40.9 (12.7) | 74.6 | LifeSense App/Passive Data Kit | Mixed sample – participants recruited via multiple settings e.g research registries. | LifeSense App/Passive Data Kit system - Passive ambulatory assessment: GPS coordinates, phone/SMS logs, duration/length, open foreground apps. | 4 months | PHQ-8, GAD-7, SPIN - week 4,7,10,13,16 |  |  |
| Osgood-Hynes et al 1998 (33) | USA | 41 | 42 (13) | 71 | COPE self-help system and Interactive Voice Response telephone calls | Mixed sample – participants recruited via multiple settings e.g advertising. | Automated Interactive Voice Response telephone calls assessing symptom severity - 6 item version of the 17 item HAM-D, PGI, WSA - and offering tailored treatment recommendations and individualised feedback. | 3 months | SCID, HAM-D, PGI, WSA - baseline, week 4, 8, 12 |  |  |
| Sharp et al 2020 (34) | USA | 28 | PA pilot: 47.4 (12.61), BC pilot: 56.4 (8.10) | PA pilot: 78.57, BC pilot: 100 | Active Living Evday Day counselling programme - 12 weekly group educational sessions about ways to increase physical activity with Fitbit/Actigraph 3GTX+ monitoring activity levels | Not reported | Fitbit/Actigraph 3GTX+ monitoring activity levels | 3 months | QIDS-SR16, SF-36, SF-36, PSQI, BFI, SHAPS, Physical Activity Stages of Change Questionnaire, Physical Activity Self-Efficacy Questionnaire - PA pilot: baseline, week 7, 13, BC pilot: baseline, week 13, 25 |  |  |
| Smit et al 2023 (45) | Netherlands | 56 | 45.9 (12.9) | 83.9 | TRANS-ID Tapering System | Mixed sample – participants recruited via multiple settings e.g advertising. Formerly depressed patients in remission. | 5x measurements per day assessing restlessness and mood for 4 months with reminder sent via text-message. SCL-90 weekly for 6 months | 5x daily assessments - 4 months, SCL-90 weekly for 6 months | SCL-90 - weekly for 6 months |  |  |
| Vachon et al 2016 (36) | France | 30 | 51.4 (9.6) | 62.5 | 2x daily self-report mood | Secondary care - outpatients | 2x daily self-report mood assessing affective/cognitive states relating to depression, visual analogue scale for depression, self-esteem, physical self, quality of life, coping, rumination, anxiety. | 5 months | MINI - fortnightly |  |  |
| Van der Watt et al 2022 (40) | South Africa | 50 (30 with Depression, 11 with Bipolar Disprder, 9 with diagnosis: Other) | 39.49  (11.17) | 74% | Weekly QIDS, ASRM administered via telephone | Secondary care - inpatients | Weekly QIDS, ASRM administered via telephone | 4 months | CTQ, LEC - baseline, CSSRS, PSQI - baseline week 4, 8, 12, 16 |  |  |
| Webb et al 2022 | USA | Total: 39, Adolescents with depression: 24 | 15.7 (1.9) | 66.7 | 12 weekly course of Behavioural Activation | Not reported | Weekly SHAPS, CES-D, BADS-SF for 15 weeks | 15 weeks | 2x 5 day ambulatory assessment periods measuring affect, probabilistic reward tasks, fMRI reward tasks |  |  |
| Xia et al 2022 (37) | USA | 41 (24 with depression) | 23.5 (3.5) | 68% | Beiwe system - GPS geolocation and accelerometer data | Secondary care - outpatients | Beiwe system - GPS geolocation and accelerometer data - continuous | 3 months | SCID-II - baseline |  |  |
| **ASRM - Altman Self-Rating Mania Scale, QIDS - Quick Inventory of Depressive Symptomatology, IDS - Inventory of Depressive Symptomatology, HAM-D - Hamilton Depression Rating Scale, CGI-BP - Clinical Global Impressions - Bipolar Scale, YMRS - Young Mania Rating Scale, HDRS-17 - Hamilton Depression Rating Scale 17, SCID - Structured Clinical Interview for DSM-IV, PSS - Perceived Stress Scale, FAST - Functional Assessment Short Test, WHOQoL-BREF - World Health Organisation Quality of Life Scale, BDI - Beck Depression Inventory, HDRS6 - 6-item Hamilton Depression Rating Scale, MARS - Medication Adherence Rating Scale, RRS - Ruminative Response Scale, PSWQ - Penn State Worry Questionnaire, BAS - Behavioural Activation Scale, VSS-A - Verona Satisfaction Scale-Affective Disorder, BAI - Beck Anxiety Inventory, ALS-18 - Affect Lability Score Short Version, Life-Rift - Level of General Functioning and Coping: Longitudinal Interval Follow-up Evaluation, BHS - Beck Hopelessness Scale, GSE - General Self-Efficacy Scale, HLOC - Health Locus of Control Scale, PICS - Perceived Involvement in Care Scales, RBANS - Repeatable Battery for the Assessment of Neuropsychological Status, NOS - Not Otherwise Specified, NIMH - National Institute of Mental Health, SMART - Specific Measurable Achievable Realistic Timebound, MADRS-S - Montgomery–Åsberg Depression Rating Scale Self-Assessment, MOS-SSS - Medical Outcomes Study Social Support Survey, GSEVDEP - Global Measure of Severity of Depression, GSEVMANIA - Global Measure of Severity of Mania, GPF - Global Measure of Psychosocial Functioning: Mania, GQOL - Global Measure of Quality of Life, SF-12 - Short Form Health Survey, Q-LES-Q - Quality of Life Enjoyment and Satisfaction Questionnaire, TIME - Time to Intervention for Mood Episode, BISS - Bipolar Inventory of Symptoms Schedule, bCBT - Blended Cognitive Behavioural Therapy, TAU - Treatment As Usual, PHQ-9 - Patient Health Questionnaire-9, QIDS-C16 - Quick Inventory of Depressive Symptomatology-16, CAGE-AID - CAGE Questionnaire Adapted to Include Drugs, PCL-C - PTSD Checklist - Civilian Version, AUDIT - Alcohol Use Disorders Identification Test, SF-36 - Short Form Health Survey - 36, FEP - Questionnaire for the Evaluation of Psychotherapeutic Progress, RSES - Rosenberg Self-Esteem Questionnaire, AEs - Adverse Events, MADRS - Montgomery-Åsberg Depression Rating Scale, GAD-7 - General Anxiety Disorder-7, QIDS-SR16 - Quick Inventory of Depressive Symptomatology - Self Report-16, ReMAP - Remote Monitoring in Psychiatry, PSQI - Pittsburgh Sleep Quality Index, IPAQ - International Physical Activity Questionnaire, ASERT - Aktibipo Self-Rating Questionnaire, EMA - Ecological Momentary Assessment, DSM-5 - Diagnostic and Statistical Manual of Mental Disorders 5th Edition, WHO-5 - World Health Organisation Five Well-Being Index, MDQ - Mood Disorder Questionnaire, HC - Healthy Controls, IDS-C - Inventory of Depressive Symptomatology Clinician Rated, DASS-21 - Depression, Anxiety and Stress Scale 21-item, BRMRS - Bech-Rafaelsen Mania Rating Scale, PLC - Personal Life Chart App, PHR-BD - Personal Health Record for Bipolar Disorder, QBL-NL - Questionnaire for Bipolar Illness Netherlands, OQ-45.2 - 45-item Outcome Questionnaire, MANSA - Manchester Short Assessment of Quality of Life, NEL - 40-item Dutch Questionnaire on Patient Empowerment, SCAN - Schedules for Clinical Assessment in Neuropsychiatry, SMS - Short Messaging Service, MINI - Mini-DIPS Neuropsychiatric Interview, PANSS - Positive and Negative Symptom Scale, SNS - Self-Evaluation of Negative Symptoms, CGI - Clinical Global Impression Scale, IAPT - Improving Access to Psychological Therapies, REDCap - Research Electronic Data Capture, WSAS - Work and Social Adjustment Scale, CESD - Center for Epidemiologic Studies Depression, PDD - Personality Dynamics Diary, BADS-SF - Behavioural Activation for Depression Scale - Short Form, BFI - Big Five Inventory, AQoL-8D - Assessment of Quality of Life, Normalised Entropy - Variance of time that a participant spent at a certain location, Location Variance - Variability in a participant’s stationary location, Number of GPS Geolocation Coordinate Clusters - Clustering technique applied to stationary states of participants, SPIN - Social Phobia Inventory, PGI - Patient Global Impression, SHAPS - Snaith-Hamilton Pleasure Scale, K-SADS - Kiddie Schedule for Affective Disorders and Schizophrenia, PSR - Psychiatric Rating Scale, IDS-SR - Inventory for Depressive Symptomatology Self Report.** | | | | | | | | | |  |  |
